# The Impact of Paediatric Obesity on Drug Pharmacokinetics: A Virtual Clinical Trials Case Study with Amlodipine

**DOI:** 10.3390/pharmaceutics16040489

**Published:** 2024-04-02

**Authors:** Khairulanwar Burhanuddin, Afzal Mohammed, Raj K. S. Badhan

**Affiliations:** School of Pharmacy, College of Health and Life Science, Aston University, Birmingham B4 7ET, UK; 149027436@aston.ac.uk (K.B.); a.u.r.mohammed@aston.ac.uk (A.M.)

**Keywords:** paediatric, obesity, pharmacokinetics, PBPK, amlodipine

## Abstract

The incidence of paediatric obesity continues to rise worldwide and contributes to a range of diseases including cardiovascular disease. Obesity in children has been shown to impact upon the plasma concentrations of various compounds, including amlodipine. Nonetheless, information on the influence of obesity on amlodipine pharmacokinetics and the need for dose adjustment has not been studied previously. This study applied the physiologically based pharmacokinetic modelling and established a paediatric obesity population to assess the impact of obesity on amlodipine pharmacokinetics in children and explore the possible dose adjustments required to reach the same plasma concentration as non-obese paediatrics. The difference in predicted maximum concentration (C_max_) and area under the curve (AUC) were significant between children with and without obesity across the age group 2 to 18 years old when a fixed-dose regimen was used. On the contrary, a weight-based dose regimen showed no difference in C_max_ between obese and non-obese from 2 to 9 years old. Thus, when a fixed-dose regimen is to be administered, a 1.25- to 1.5-fold increase in dose is required in obese children to achieve the same C_max_ concentration as non-obese children, specifically for children aged 5 years and above.

## 1. Introduction

The prevalence of paediatric obesity worldwide has risen by approximately 20% over the past few decades, and latest reports predict that this number would double globally by 2035, affecting 208 million boys and 175 million girls [1]. The trend can be seen in developed countries, such as the United Kingdom, where obesity among children aged 4 to 6 increased by 4.5% between 2019–2020 and 2020–2021, while the percentage decreased by 2.1% to 4.3% between 2020–2021 and 2021–2022 [2,3]. The pattern has been stagnant in some parts of Europe and high-income English-speaking countries; however, the rise in the childhood obesity phenomenon has accelerated in East, South, and Southeast Asia [4].

Obesity is known to cause physiological alteration in drug distribution and elimination due to increased tissue volume, altered tissue composition, change in blood protein proportions, metabolism enzyme activity, and glomerular filtration rate (GFR) [5,6,7,8,9,10,11]. The complexity becomes even more intricate in paediatric populations due to the interplay of age-related ontogeny and obesity-related factors. The Centers for Disease Control and Prevention (CDC) defined childhood obesity as children with a body mass index (BMI) above the 95th percentile, while the World Health Organization (WHO) set obesity at +3 standard deviations (SDs) and +2 SDs from the median line for 0 to 5 years old and 5 to 18 years old, respectively [12,13].

The primary physiological changes observed in obese children are the physical attributes, namely, weight and height, which are the foundation of BMI classification for obesity. The increase in body weight relates mainly to the rise in total body fat and, to some extent, lean body mass, which impacts the volume of distribution (V_ss_) of drugs, depending on their lipophilicity and hydrophilicity [14]. Additionally, the composition of plasma components like serum albumin and α1-acid glycoprotein (AGP), essential for drugs’ protein binding, also affects the V_ss_. Notably, it has been reported that there is no difference in these plasma components between obese and non-obese children [11,15].

Drug clearance relates mainly to hepatic metabolism and renal functions. Information regarding the difference in metabolism enzyme abundance between paediatric cases with and without obesity is scarce [11]. Nevertheless, the presence of a larger liver size and higher blood flow to the liver in obese children is expected to impact hepatic clearance [11]. As for renal function, the paediatric obesity population tend to have an elevated GFR, which can alter the clearance of drugs, particularly drugs that are predominantly eliminated through renal pathways [10,16].

Dosing guidelines for obese paediatrics are typically derived from obese adjusts, and complexities in both weight dosing methodologies can contribute to non-optimal doses. Current dosing approaches in paediatric obesity have highlighted that over 60% of drugs administered to obese children elicit plasma concentrations outside of the therapeutic range and display clinically significant alterations in pharmacokinetics [17].

An aspect of this non-optimal dosing stems from the appropriate use of body weight and the correct use of body weight in anthropometrics-based dosing approaches. Typically, this involves the use of total body weight (TBW) and other methods, such as an allometric scale and dosing recommendation, which are derived from pharmacokinetic data in non-obese adults or children [18]. Other approaches have been proposed utilising body surface area (BSA), ideal body weight (IBW), and lead body weight (LBW) methods. However, given that these calculations typically invoke the use of a height component, obesity presents challenges given the normal linear growth of children can be affected [18,19,20].

Given the various physiological changes occurring longitudinally with ageing across the paediatric spectrum, in addition to the differences in physiologies specific to obese vs. non-obese children, dosing approaches based on the holistic consideration of these physiological changes in the drug pharmacokinetics have gained some traction in adults and, more recently, children. Physiologically based pharmacokinetic (PBPK) modelling, an advanced quantitative approach, helps to understand drug disposition even with the lack of concentration data and offers a promising avenue for determining optimal dosing regimens in the paediatric obesity population, and the concept has been implemented for compounds such as metformin, midazolam, clindamycin, trimethoprim, sulfamethoxazole, fentanyl, and methadone [11,21,22].

Childhood obesity contributes to various metabolic and cardiovascular complications and has profoundly changed the frequency of primary hypertension in children, with only 15% in 1988 rising to 90% in 2010 [23]. Calcium channel blockers (CCBs), such as amlodipine, felodipine, and nifedipine, are among the antihypertensive agents recommended as first-line therapy [24,25]. A study by Hanafy et al. (2009) [26] reported that obese children exhibit a significantly lower response to CCBs, including amlodipine, in terms of reducing systolic blood pressure and response rate compared to non-obese children.

Using amlodipine as a case study, this study describes the approach to develop a physiological obesity model to support pharmacokinetic-based dose optimisation for the first time in paediatric obesity populations. By utilising PBPK advancement, a robust paediatric obesity population model and amlodipine pharmacokinetic model were established, significantly impacting paediatric pharmacotherapy, filling the knowledge gap of drug disposition in this unique population and facilitating the design of personalised dosing strategies. Moreover, the insights gained may serve as a model for pharmacokinetic studies in other medications used in paediatric obesity.

The primary objectives of this study are to use the principle of mechanistic pharmacokinetic modelling and virtual clinical trials to (1) develop and validate a paediatric obesity population model, (2) address the impact of obesity on amlodipine pharmacokinetics in paediatrics, and (3) determine the dose adjustment needed for amlodipine in obese paediatric populations.

## 2. Materials and Methods

**Figure 1:**
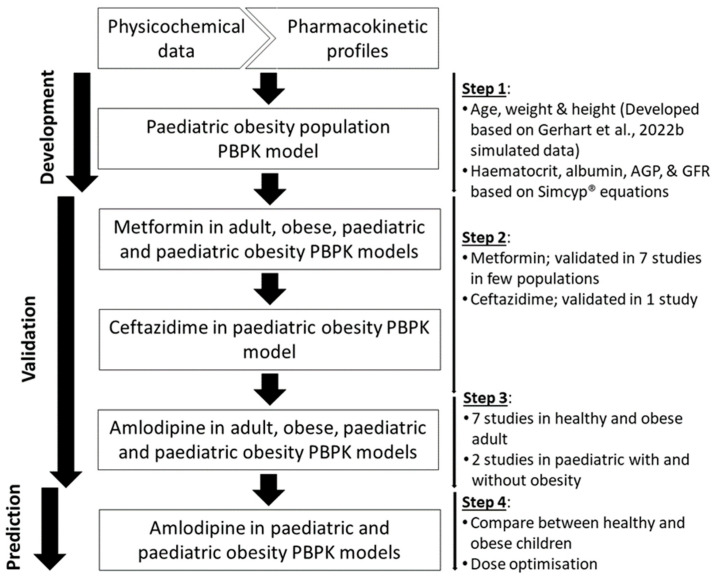
A 4-stage workflow was implemented to develop, verify, and explore the amlodipine dose in the paediatric population [11].

The PBPK modelling software Simcyp^®^ (Simcyp Ltd., a Certara company, Sheffield, UK, Version 21) was used to develop a paediatric obesity population group and to assess the optimum dose of amlodipine in the paediatric obesity population using virtual pharmacokinetic studies. We applied a workflow model with 4 stages for this study (Figure 1).

### 2.1. Step 1: Development of the Paediatric Obesity Population

For the development of the model, we focused on 6 physiological parameters that have been reported to change in obese children when compared to non-obese children, namely: (1) weight, (2) height, (3) haematocrit, (4) serum albumin, (5) AGP, and (6) estimated glomerular filtration rate (eGFR). We used published data with the primary reference for the simulated age, weight, and height shared by the Gerhart group to develop the population group [11].

#### 2.1.1. Age, Weight, and Height Relationship

The weight, height, and age correlations for paediatric obesity published by the WHO [12] and CDC [13] were the primary guidance in constructing the weight–age, height–age, and weight–height relationships for the paediatric obesity population. The definition by the WHO and CDC was used to develop weight curves. Furthermore, we refined the curve based on the paediatric obesity population developed by Gerhart et al. (2022) [11].

According to the WHO, the definition of obese for 0 to 5 years old is +3 SDs from the median line of the BMI-for-age and weight-for-height curves, while for 5 to 18 years old, obese is defined as BMI above +2 SDs from the median line of the BMI-for-age curve [12,27,28]. As for the CDC, child obesity is defined as a BMI range above the 95th percentile or greater based on the BMI-for-age curve [13,29]. Additionally, we used the 95th percentile of weight-for-length data published by the CDC for 0 to 5 years old to validate the simulated weight-for-height curve [30].

#### 2.1.2. Haematocrit–Age Relationship

Reported changes in haematocrit are conflicting within the literature. Several publications reported no significant difference between healthy and obese children as well as between genders [11,31,32,33], whilst others contradict these findings [34,35]. Furthermore, a study by Belo et al. (2014) [36] reported significant differences between genders as well as obese and non-obese paediatrics for males but not for females. Considering all these reports, no additional change was made to the haematocrit in paediatric obesity.

#### 2.1.3. Protein-Binding-to-Age Relationship

No significant difference was reported in serum albumin value between paediatrics with and without obesity by several publications [11,15,37,38,39] except for 2 studies by Marginean et al. published in 2014 and 2016 [40,41]. Furthermore, no specific correlation has been reported in terms of the albumin-to-age relationship [11]. Similarly, a significant difference in AGP was reported between obese and non-obese children as well as between genders by Sobieska et al. (2013) [42], Gibson et al. (2014) [43], and Ferrari et al. (2015) [44], but not by Gerhart et al. (2022) [11]. Since conflicting results were reported in both albumin and AGP, the default equations in Simcyp^®^ for both albumin and AGP in paediatrics were utilised for simulation and compared to all published data.

#### 2.1.4. Glomerular Filtration Rate (GFR)-to-Age Relationship

The GFR is an established measure of renal function. The GFR values for paediatric obesity were reported in 3 publications, namely, Duzova et al. (2013) [45], Goknar et al. (2015) [16], and Correia-Costa et al. (2016) [10], which were used to validate the predicted GFR from the model.

### 2.2. Step 2: Validation of a Paediatric Obesity Population with Metformin and Ceftazidime Compound Files

#### 2.2.1. Step 2.1: Validation with Metformin

A previously developed and fully validated metformin compound file has been reported in the literature and incorporated into the Simcyp^®^ compound library [46]. The compound was utilised in the paediatric obesity model with some adaptions, namely the fraction of dose absorbed (fa) and V_ss_ parameters (Table 1). A revised fa was fitted based on several publications [47,48]. The V_ss_ was estimated using the Rodgers and Rowland approach [49,50]. The estimated V_ss_ value correlates with several published studies [47,51].

Since several changes were made to the metformin compound file, the adapted metformin model was verified in healthy adults, obese adults, and paediatric populations, followed by validation of the paediatric obesity population model with the validated metformin model. All the validations were confirmed with observed data from 7 studies (Table 2). All virtual clinical trial simulations were run with a 10 × 10 design (10 trials with 10 subjects per trial), where the dosage regimen, age range, and male-to-female ratio were comparable to the published studies (Table 2).

#### 2.2.2. Step 2.2: Validation with Ceftazidime

A previously published ceftazidime compound (Table 3) was further used to validate the paediatric obesity population model [59]. Several publications have previously validated the ceftazidime compound file in healthy adult and paediatric populations [59,60,61,62]. Therefore, the validated ceftazidime compound file was used to validate the paediatric obesity population model using sparse ceftazidime concentration data reported by Maharaj et al. (2021) [58] (Table 2).

Although the observed pharmacokinetic data did not differentiate between obese and non-obese subjects, most subjects were classified as obese, which justified using the pharmacokinetic data for paediatric obesity population validation. Simulations were conducted on median, highest, and lowest doses with a 10 × 10 study design. The age and male-to-female ratio corresponded to the published data. Virtual paediatric obese subjects administered with more than 2 g/dose in the simulation were excluded from the predicted mean concentration–time profile.

### 2.3. Step 3: Verification with Amlodipine

Physiochemical and pharmacokinetic parameters describing the amlodipine model utilised in this study were obtained and adapted from several publications [63,64,65] (Table 4). For the distribution model, we utilised a full-body PBPK model with the V_ss,_ which was estimated using the Rodgers and Rowland approach based on the tissue partition coefficients (Kp) [49,50]. The Kp value was predicted by fitting the simulated with the observed plasma concentrations, with the resulting V_ss_ correlating with that published [66].

Renal function was determined firstly by scaling kidney weight in adults (~317 g) based on correlations incorporating body weight [70] with glomerular filtration rate calculated using the Cockcroft and Gault equations [70]. Ghobadi et al. reported that kidney size showed a similar increase in relation to variations in the body mass index (BMI) and body surface area (BSA), hence a separate model was not required to be developed for obesity populations and therefore paediatric GFR was simulated using the modification of diet in renal disease (MDRD) equation [71].

The intrinsic clearance by CYP3A4 liver enzymes [68] and human intestinal microsome [69], utilised within the model, and all the adapted parameters were validated with observed data from 6 studies involving healthy adults, 1 study with obese adults, 1 study with paediatrics, and 1 study involving paediatrics both with and without obesity (Table 5). The virtual clinical trials were run with a 10 × 10 design with the dosing regimen, male-to-female ratio, and age range corresponding to the published studies. For the obese adult population, simulations were performed with 200 subjects taking amlodipine 5 mg and 10 mg daily for 28 days with a 1:1 ratio, given the publication reported the parameters with limited information on the dose taken by the obese subjects.

For comparison with the observed trough concentration (C_min_) in the paediatric population [72], virtual trials in the paediatric population matching the demographic of observed data were run at 3 doses daily for 21 days, with the median (0.15 mg/kg/day), 1st interquartile (0.10 mg/kg/day), and 3rd interquartile (0.22 mg/kg/day). Additionally, the virtual paediatric subjects administered with more than 5mg/day were filtered out following the maximum daily dose allowed, resulting in 99%, 85%, and 58% of the virtual subjects being included for verification for 0.10, 0.15, and 0.22 mg/kg/day doses, respectively.

For verification in the paediatric obesity population, a study by Flynn et al. (2006) [73], who reported concentration profiles for paediatrics with 43.2% of the children categorised as obese, was utilised. We ran the virtual trials with a 20 × 10 design with a ratio of 50:50 for the male-to-female and obese-to-non-obese paediatric subjects. The simulations were made with once- and twice-daily doses at 3 dose levels for 28 days, 0.03 mg/kg/day (minimum), 0.17 mg/kg/day (mean), and 0.77 mg/kg/day (maximum), with the absolute doses of 1.3 mg/day and 20 mg/day. With the cap, the percentage of simulated obese children included for amlodipine model verification in once-daily and twice-daily dosing was 50.73% and 50.86%, respectively.

**Table 5:** Validation datasets used for verification of the amlodipine model.

Reference	Subjects	Age (Years)	Dose Regimen	PK Sampling Duration
Healthy subjects				
[74]	Single dose: 12 healthy males Multiple doses: 56 healthy males	Single dose: 25.8 ± 3.8 Multiple dose: 26.1 ± 36	Single-dose fasting: 10 mg intravenous (1 mg/min) in period 1, 34-day washout period, 10 mg oral dose (2–5 mg capsule) Multiple doses: 15 mg once daily (3 × 5 mg capsule) or placebo for 14 days	Single dose: Up to 144 h post-dose Multiple doses: Day 1: up to 24 h post-dose, Day 7: pre-dose and up to 14 h post-dose, Day 14: up to 168 h post-dose
[67]	12 healthy males	23–34	2.5 mg single dose 5 mg single dose 10 mg single dose With 14-day washout period between each dose	Up to 144 h post-dose
[66]	13 patients with hypertension (10 males, 3 females)	28–45	1st dose of 10 mg intravenously, after Day 4 of the intravenous dose followed by 2.5 mg oral once daily for 10 days	After 10 days of amlodipine dose, up to 24 h post-dose
[75]	12 healthy subjects (7 males, 5 females)	46–76	5 mg oral once daily for 14 days	Up to 48 h post-dose after the 1st dose and after the last dose at 14 days
[76]	24 healthy subjects	Adult	10 mg oral once	Up to 72 h post-dose
[77]	28 patients with hypertension (10 males, 18 females) BMI = 30.6 ± 1.3	22–50	5 mg oral once daily for 8 weeks	After the 1st dose, up to 24 h post-dose After the last dose, up to 240 h
Obese subjects				
[78]	22 hypertensive patients: - 4 normal - 6 overweight - 12 obese - 27.3% male	16 adults (<65 years old with majority 50–60 years old) 6 elderly (≥65 years old)	Fixed-dose combination of telmisartan and amlodipine once daily: 40/5 mg—8 subjects 80/5 mg—6 subjects 80/10 mg—8 subjects	Up to 72 h post-dose at steady state
Paediatric subjects				
[72]	9 (6 males, 3 females)	0.5–12	0.15 (0.10–0.22) ^a^ mg/kg/day (oral solution)	Sparse trough concentrations
Mixture of paediatric with and without obesity				
[73]	73 (49 males, 24 females) - 43.2% obese children	1.0–17.7	0.17 ± 0.13 (0.03–0.77) mg/kg/day - Absolute dose: 1.3–20 mg/day - Administered either once or twice daily (tablet and suspension)	Sparse samples

BMI, body mass index (kg/m^2^); Mean ± SD; (n–n), range; ^a^ median (interquartile range); PK, pharmacokinetic.

### 2.4. Step 4: Influence of Obesity on Amlodipine Pharmacokinetic Parameters and Dose Adjustment in the Paediatric Obesity Population

Following validation in the paediatric obesity population, we explored the impact of obesity on the pharmacokinetic and plasma concentrations of amlodipine. A 10 × 10 trial design for 3 different age groups in paediatrics both with and without obesity was set as follows: (i) 2 to 6 years old, (ii) 6.01 to 12 years old, and (iii) 12.01 to 18 years old.

Each group was dosed with amlodipine at a dose of 2.5 mg, 5 mg, and 10 mg once daily for 3 weeks, except for group 1 (2 to 6 years old), in which we simulated the virtual subjects to be administered 0.20 mg/kg daily and 2.5 mg once daily for every 3 weeks. The dose selection for simulation was based on the recommended minimum and maximum dose for children based on age group. In the 2 to 6 years old group, the dose selected was the maximum starting dose for the weight-based dose and for the 6 years and above group it was the minimum dose for the fixed dose [79,80].

In order to assess the need for dose adjustment, the amlodipine therapeutic window of 1 ng/mL to 57.2 ng/mL and the toxic level of 67 ng/mL were used as a general guide to ensure the adjusted doses yield concentrations within the safe window [73,81,82,83]. To simulate the amlodipine peak concentrations at a steady state (C_max_), virtual clinical trials in paediatric obesity were performed with a 10 × 10 design for the following age groups: (i) 2 to 4 years old, (ii) 4.01 to 6 years old, (iii) 6.01 to 8 years old, (iv) 8.01 to 10 years old, (v) 10.01 to 12 years old, (vi) 12.01 to 14 years old, (vii) 14.01 to 16 years old, and (viii) 16.01 to 18 years old.

In each virtual clinical trial, amlodipine was administered over 2-week periods at varying fixed doses starting from 2.5 to 10 mg daily. In addition, for the age group from 2 to 12 years old, simulated weight-based dosages ranging from 0.10 mg/kg to 0.40 mg/kg per day were performed. The primary objective was to attain a comparable simulated C_max_ at steady state in healthy paediatrics administered with fixed daily doses of 2.5 mg and 5 mg, alongside weight-based doses of 0.10 mg/kg and 0.40 mg/kg.

The simulated minimum and maximum dose ranges selected for the simulation were based on the British National Formulary for Children (BNFc) and amlodipine product insert [79,80].

### 2.5. Prediction Performance

For the validation of all the simulated physiological parameters, we adopted a visual predictive checking (VPC) strategy to validate the predicted values. The method was explained at the 2012 United States Food and Drug Administration Paediatric Advisory Committee [84] and was widely used to develop population models [85,86]. Validation by the VPC approach was by carried out presenting the predicted and observed values with mean and SD graphically in the same graph. Most observed data points should overlap with the simulated values to be considered acceptable. As for the pharmacokinetics profile predictions, we used the VPC strategy and 2-fold (0.5–2-fold) predicted/observed ratio rules to represent the predictive performance as “optimal” unless otherwise explained [87,88,89]. This strategy was used for validation in steps 2 and 3 when comparing the predicted and observed values. As for the VPC, the simulated profiles were considered acceptable when the reported profiles overlapped within the 5th and 95th percentiles of the predicted mean concentration profiles.

### 2.6. Data and Statistical Analysis

We extracted all the population validation and compound data using WebPlotDigitizer version 4.5 (https://apps.automeris.io/wpd/) (accessed on 23 July 2023). We performed statistical analysis using a non-parametric, unpaired Student’s *t*-test to compare the simulated amlodipine pharmacokinetic parameters between healthy and obese children in step 4. The significance test was performed with *p* < 0.05. The statistical analysis was run using GraphPad Prism Version 8 for Windows (GraphPad Software, La Jolla, CA, USA).

## 3. Results

### 3.1. Step 1: Development of the Paediatric Obesity Population

The relevant published data for all physiological parameters, including height, weight, haematocrit, serum albumin, AGP, and GFR, were within the range of individual prediction values, which validated the paediatric obesity population. The results are detailed in the Supplementary Materials (Section 1).

### 3.2. Step 2: Validation of the Paediatric Obesity Population

#### 3.2.1. Step 2.1: Validation with Metformin

All predicted pharmacokinetic parameters, namely, C_max_, maximum concentration at steady state (C_maxss_) area under the curve to time (AUC_0-t_)_,_ area under the curve to time at steady state (AUC_0-tss_), time to reach maximum concentration (T_max_)_,_ and oral clearance (CL/F), were within 0.75- to 1.5-fold of the observed parameters reported in publications (Table 6). In addition, the BMI (kg/m^2^) distribution for the simulated obese adult population was comparable with the observed study population (40.5 ± 6.9 vs. 39.5 ± 5.13) [54].

**Figure 2:**
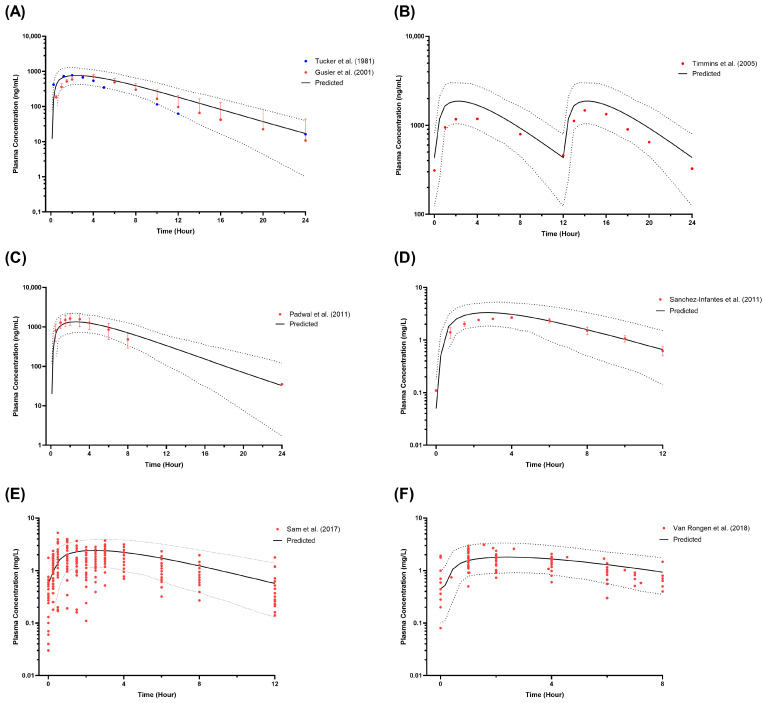
Simulated metformin plasma concentration in healthy adults (**A**,**B**), obese adults (**C**), paediatrics (**D**), and paediatric obesity (**E**,**F**). (**A**) Single-dose 500 mg in healthy adults [47,52]; (**B**) Multiple-dose 1000 mg twice daily in healthy adults [53]; (**C**) Single-dose 1000 mg in obese adults [54]; (**D**) Multiple-dose 850 mg once daily in paediatric population [55]; (**E**) Multiple-dose 1000 mg twice daily in paediatric obesity to match Sam et al. (2017) [57] subjects’ demographic; (**F**) Multiple-dose 1000 mg twice daily in paediatric obesity to match Van Rongen et al. (2018) [56] subjects’ demographic; Solid lines represent the predicted mean concentration–time profile, with dotted lines representing the 5th and 95th percentile ranges; Solid circles represent individual observed data from each study. Solid circles with error bars represent the mean and SD of the observed data from each study.

Moreover, the observed profiles from all studies listed in Table 2 agree with the simulated profile based on the VPC acceptance criteria, where the published profiles fit within the 5th and 95th percentiles of the predicted plasma concentration profile, thereby confirming the adaptation of the metformin model (Figure 2A–D). As for the paediatric obesity plasma concentration profiles, the individual reported plasma concentration profiles of metformin for both published multiple-dose studies were centred around the mean simulated metformin plasma concentration (Figure 2E,F).

#### 3.2.2. Step 2.2: Validation with Ceftazidime

Furthermore, we validated the paediatric obesity population with the ceftazidime model and plasma concentration data published by Maharaj et al. (2021) [58]. Following filtering virtual subjects administered with more than 2 g/dose, the percentages of virtual subjects used for the simulated mean concentration–time profile for 16.5 mg/kg q8h, 33.8 mg/kg q8h, and 92.9 mg/kg q8h are 100%, 53%, and 4%, respectively.

**Figure 3:**
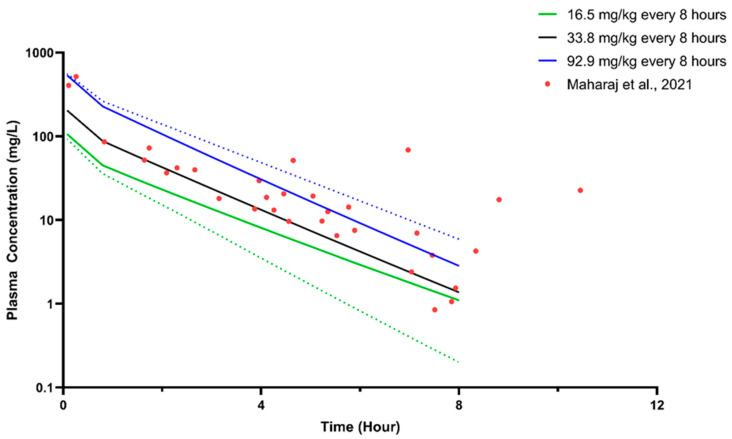
Simulated steady-state plasma concentration of ceftazidime for the paediatric population. Solid lines represent the predicted mean concentration–time profile, with dotted lines representing the 5th and 95th percentile ranges. Solid circles represent the mean of the observed clinical data from Maharaj et al. (2021) [58].

Considering only the sparse plasma concentration of ceftazidime available for validation of the paediatric obese population, we only used the VPC method, where a majority (84%) of the observed concentration data fell within the 5th and 95th percentile of the simulated concentration profile (Figure 3). Furthermore, the percentage of concentrations within the acceptance limit was comparable with the number of obese subjects recruited in the study (84.00% vs. 82.80%), thus verifying the paediatric obesity population model.

### 3.3. Step 3: Verification of the Amlodipine Model

Predicted pharmacokinetic parameters for adults and obese adults in single- and multiple-dose studies, including C_max_, AUC_0-t_, AUC_0-inf,_ C_maxss_, AUC_0-tss,_ AUC_0-infss_, and T_max,_ were within 0.5–2-fold of the reported data except for AUC_0-infss_ for the multiple-dose study by Bainbridge et al. (1993) [75] (Table 7). Since only pharmacokinetic parameters were revealed for the obese adult population, verification of the amlodipine model in obese adults was based on the comparison between predicted and observed parameters.

**Figure 4:**
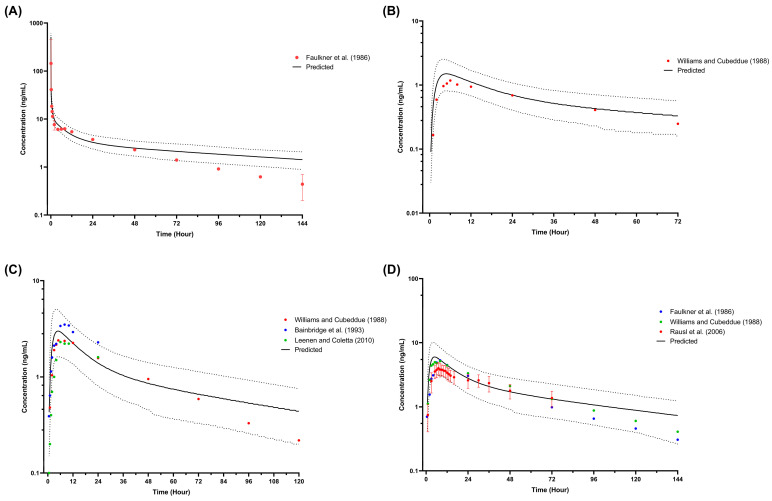
Simulated plasma concentration of amlodipine single dose in healthy adults. (**A**) A 10 mg intravenous single dose [74]; (**B**) 2.5 mg oral single dose [67]; (**C**) 5 mg oral single dose [67,75,77]; (**D**) 10 mg oral single dose [67,74,76]. Solid lines represent the predicted mean concentration–time profile, with dotted lines representing the 5th and 95th percentile ranges. Solid circles represent observed clinical data from each study. Solid circles with error bars represent the mean and range for Faulkner et al. (1986) [74] and the mean and SD of the observed clinical data for Rausl et al. (2006) [76].

**Figure 5:**
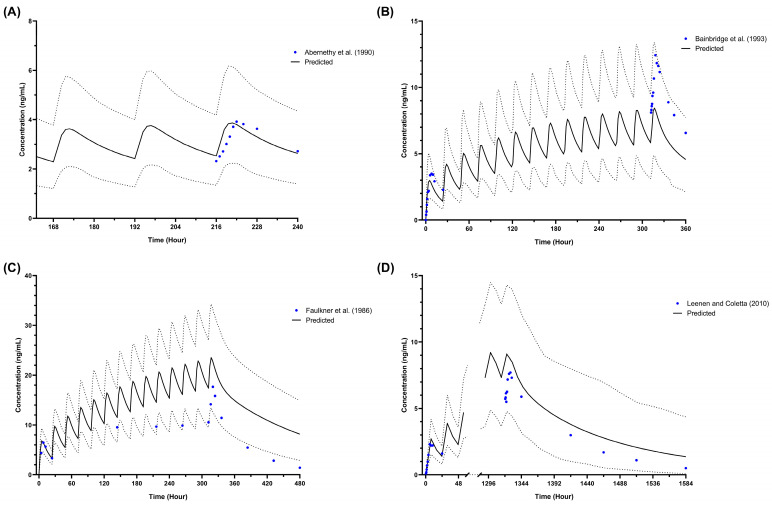
Simulated plasma concentration of amlodipine multiple dose in healthy adults. (**A**) A 2.5 mg daily dose for 14 days [66]; (**B**) 5 mg daily for 14 days [75]; (**C**) 15 mg daily for 14 days [74]; (**D**) 5 mg daily for 8 weeks [76]. Solid lines represent the predicted mean concentration–time profile, with dotted lines representing the 5th and 95th percentile ranges. Solid circles represent observed clinical data from each study.

Observed plasma concentrations for both single-dose and multiple-dose studies in adults concurred with the simulated profiles and fit within the 5th and 95th percentiles (Figure 4 and Figure 5). Among the 12 observed profiles, only 2 fell outside the defined acceptance range at the elimination phase, precisely the last 3 points. These instances were associated with amlodipine administered intravenously at a 10 mg single dose (Figure 4A) and orally at 15 mg daily for 14 days (Figure 5C). In addition, the profiles at steady state were underpredicted and overpredicted when simulated with once-daily doses of 5 mg and 15 mg for 14 days, respectively (Figure 5B,C).

**Figure 6:**
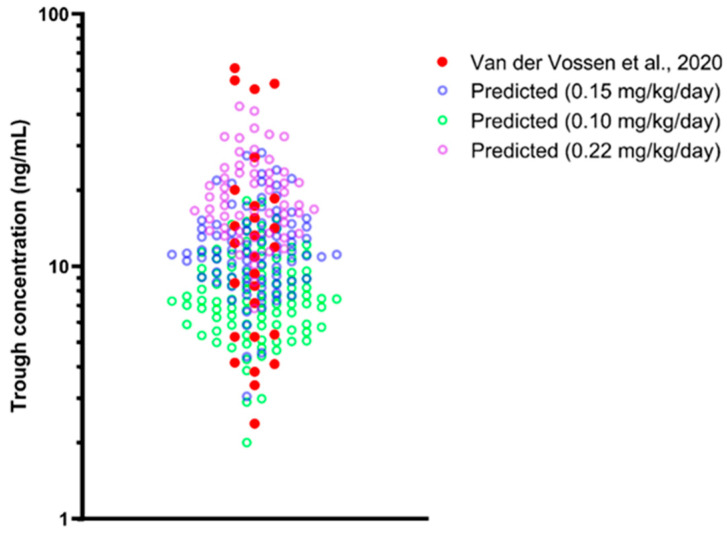
Simulated steady-state trough concentration (C_min_) of amlodipine multiple dose in the paediatric population. Solid red circles represent the observed trough concentration of amlodipine published by van der Vossen et al. (2020) [72]. Coloured open circles represent predicted trough concentration for 3 doses: 0.15 mg/kg/day, 0.10 mg/kg/day, and 0.22 mg/kg/day.

For simulations of paediatric C_min_, the predicted amlodipine residual concentrations are well within the range of observed amlodipine residual concentrations, with four observed samples above the highest predicted C_min_ (Figure 6)_._

**Figure 7:**
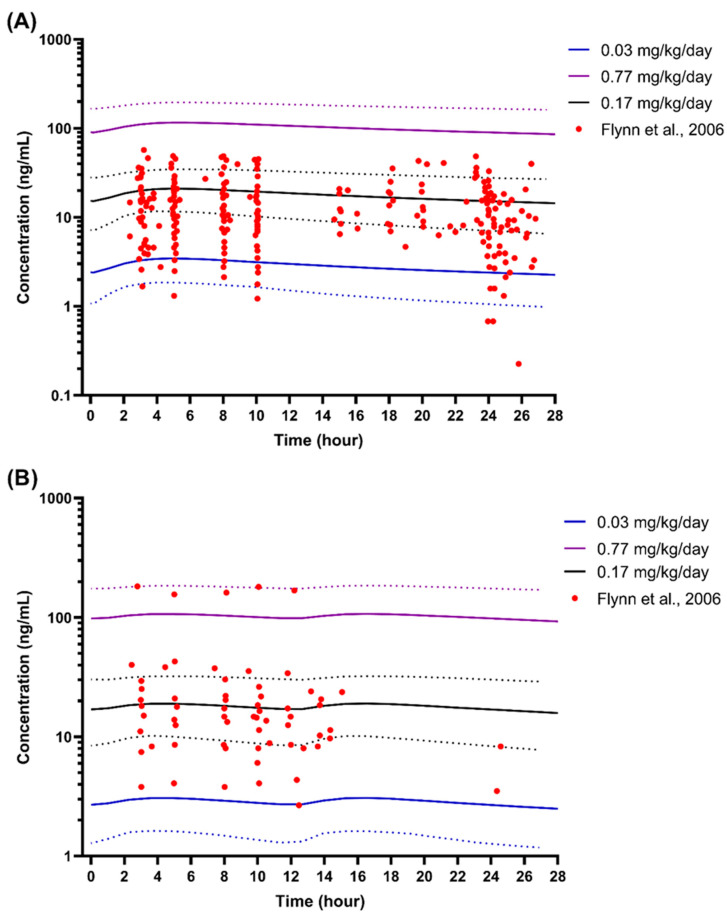
Simulated plasma concentration of amlodipine in paediatrics with and without obesity. (**A**) Once daily dose at steady state; (**B**) Twice daily dose at steady state; Solid lines represent the predicted mean concentration–time profile, with dotted lines representing the 5th and 95th percentile ranges. Solid circles represent observed plasma concentrations from Flynn et al. (2006) [73].

Verification of the amlodipine model in the paediatric obesity populations showed that all the observed plasma concentrations overlapped within the 5th and 95th percentiles of the minimum and maximum daily doses, except for six observed concentrations in the once-daily dose profile (Figure 7). Moreover, most observed plasma concentrations spread within the simulated mean dose plasma concentration profiles (Figure 7), thus validating the amlodipine model in obese children.

### 3.4. Step 4: Impact of Paediatric Obesity on Amlodipine Pharmacokinetics

#### 3.4.1. Comparison of Non-Obese and Obese Paediatrics

**Figure 8:**
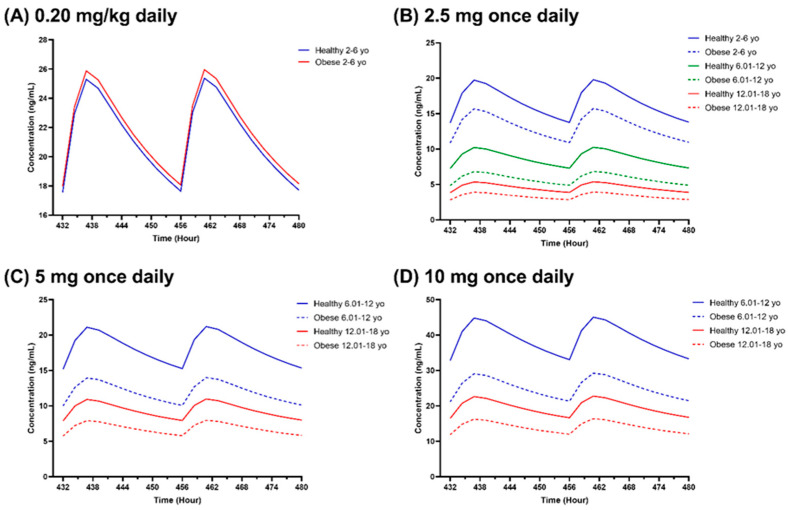
Simulated steady-state amlodipine plasma concentration profiles in healthy and obese children for age groups of 2 to 6 years old, 6.01 to 12 years old, and 12.01 to 18 years old. (**A**) A 0.20 mg/kg daily dose; (**B**) 2.5 mg once daily; (**C**) 5 mg once daily; (**D**) 10 mg once daily. In graph (**A**), different colours represent paediatrics with and without obesity in the 2 to 6 age group. The dotted lines in the (**B**–**D**) graphs represent obese paediatric populations. Different coloured lines in the (**B**–**D**) profiles represent different age groups.

**Figure 9:**
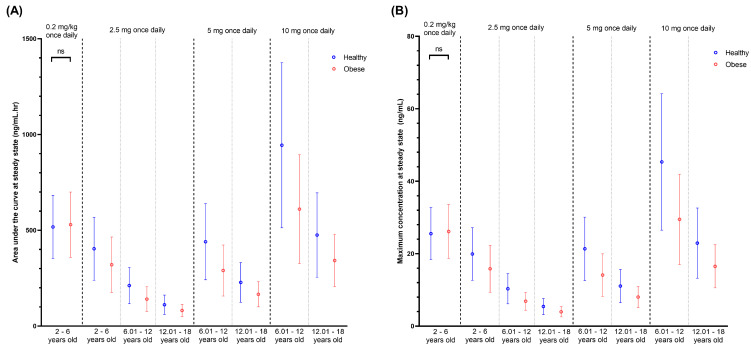
Predicted AUC (**A**) and C_max_ (**B**) at steady-state for healthy and obese paediatrics for 4 doses and 3 different age groups. The coloured circles represent the mean, and the horizontal lines represent the standard deviations. “ns”, *p* > 0.05.

An approximately 2-fold decrease was observed in the simulated steady-state plasma concentration profiles (Figure 8), AUC, and C_max_ (Figure 9) as the age group increased. Compared to the non-obese children, the AUC and C_max_ of obese children decreased by 35.30% and 20.49%, respectively (Figure 9). Additionally, the comparison of AUC and C_max_ showed a statistically significant difference between obese and non-obese paediatrics for all age groups when administered amlodipine as a fixed dose (Figure 9). In contrast, the difference was insignificant when amlodipine was dosed based on TBW in the 2 to 6 years old age group (Figure 9). For comparison of clearance and V_ss_, statistically significant differences were noted for both fixed and weight-based dose regimens as well as all age groups (Figure S9).

#### 3.4.2. Dose Adjustments in Paediatric Obesity

**Figure 10:**
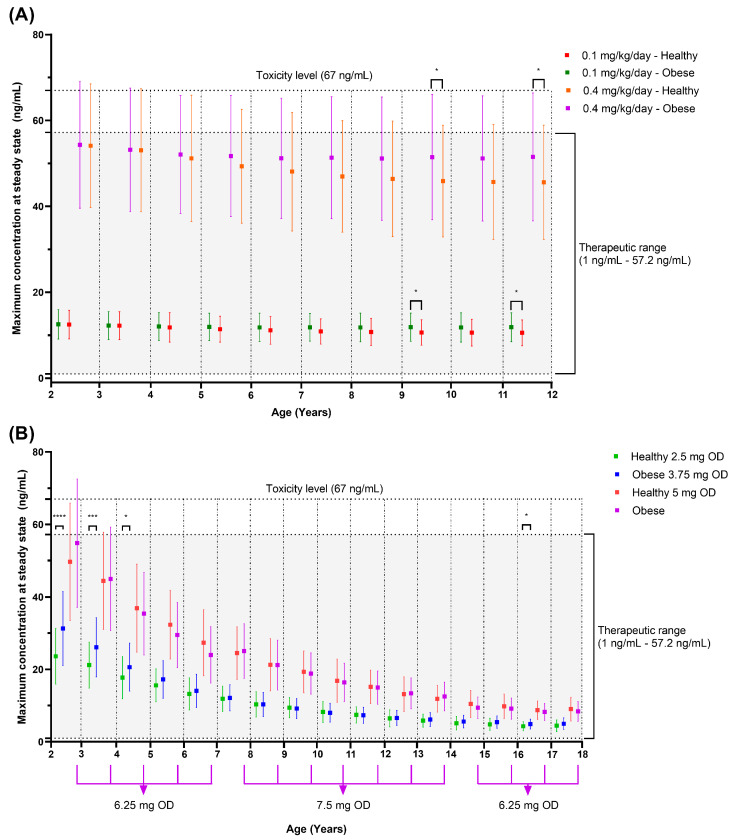
Predicted C_max_ in obese and non-obese paediatrics with weight-based dose (**A**) and fixed-dose (**B**) approaches for dose adjustment. The horizontal lines represent the mean and standard deviations. For fixed doses, 6.25 mg OD and 7.5 mg OD are suitable in obese children, depending on the age range, to match the healthy children’s 5 mg dose, as shown by the purple lines under the x-axis. OD, once daily; ****, *p* < 0.0001; ***, *p* < 0.001; *, *p* < 0.05.

Our simulations showed that weight-based doses resulted in comparable predicted C_max_ in children both with and without obesity except for ages 9.01–10 years (0.1 mg/kg/day: *p* = 0.0453, 0.4 mg/kg/day: *p* = 0.0405) and 11.01–12 years (0.1 mg/kg/day: *p* = 0.0380, 0.4 mg/kg/day: *p* = 0.0335), where the differences were statistically significant (Figure 10A). None of the simulated C_max_ values exceeded the maximum therapeutic concentration (57.2 ng/mL) and toxicity level (67 ng/mL) in both populations when dosed with a 0.10 mg/kg amlodipine starting dose (Figure 10A). The same was seen for the daily dose, not one simulated subject was administered above the maximum daily dose of 10 mg as recommended by the BNFc [79].

In contrast, the maximum dose of 0.40 mg/kg resulted in 7.55% and 1.92% of healthy and obese paediatrics as early as 5.01–6 years and 3.01–4 years old being dosed above 10 mg daily, respectively. The proportion reached 100% for the age group 7.01–8 years old in the paediatric obesity group and more than 90% for the 10.01–11 years old group in the non-obese paediatric population (Figures S13 and S14).

For the predicted C_max_, the percentage that tops the toxicity level (67 ng/mL) was less than 20% for obese children across the age range up to 12 years (Figure S14). On the other hand, for non-obese paediatrics, the percentage of C_max_ that exceeded 67 ng/mL was more than 20% in the age group 2–4 years (Figure S13). Generally, the value of predicted C_max_ that falls above the maximum therapeutic concentration of 57.2 ng/mL was 28.30% to 38.30% in obese paediatrics and 19.15% to 37.5% in non-obese children, depending on the age group (Figure 10A).

For fixed-dose simulations, an approximately 1.25 to 1.5 times higher dose is needed in obese children in order to achieve the same C_max_ as non-obese children (Figure 10B). A significant difference in C_max_ was seen in the younger age group (2 to 5 years old) even after a 1.5-fold increase in the starting dose in obese children compared to 2.5 mg daily in non-obese paediatrics. Another notable difference at the starting dose was seen in the 16 to 17 years old group (Figure 10B). As for the maintenance dose of 5 mg daily, the 1.25- to 1.5-fold dose increase in obese paediatrics resulted in comparable C_max_ to the non-obese paediatric populations (Figure 10B). A similar trend is seen with higher maintenance doses. The increment was set based on the dose in tablet form available in the market [79]. As for the C_max_, the lower age groups were more at risk of concentrations above the therapeutic and toxicity range than the higher age groups with both weight-based and fixed doses (Figure 10).

## 4. Discussion

Paediatric obesity is an epidemic which is increasing worldwide, with reports predicting that this number would double globally by 2035, affecting 208 million boys and 175 million girls [1]. Associated with this is a range of physiological alterations in drug distribution and elimination, as a result of increased tissue volume, altered tissue composition, change in blood protein proportions, metabolism enzyme activity, and glomerular filtration rate (GFR) [5,6,7,8,9,10,11].

The rising trend of obesity among children profoundly impacts the prevalence of hypertension in childhood, and amlodipine as a CCB is recommended as treatment when it is unresponsive to lifestyle modification [12,23,73,90,91,92,93].

Physiological changes due to obesity, which influence the distribution and elimination process of drugs, might impact the pharmacokinetics and consequently affect the amlodipine response [94]. A study by Hanafy et al. (2009) [26] reported that the effect of CCBs, including amlodipine, in reducing systolic blood pressure and the percentage of response to CCB treatment was significantly lower in obese children when compared to non-obese children. However, the contribution from a pharmacokinetic perspective on the substantially lower efficacy in the paediatric obesity population is lacking due to the paucity of published amlodipine plasma concentration data in the population group.

The application of robust and validated physiologically based pharmacokinetic modelling permits analysis of the influence of obesity on the drug’s pharmacokinetics and exploration for a pragmatic recommendation of the optimum dose [21,95]. Therefore, we have adapted the concept, developed a virtual paediatric obesity population, and used it to explore the pharmacokinetic differences and find the optimum dosing strategy for amlodipine in children with obesity using this mechanistic modelling.

### 4.1. Step 1: Development of the Paediatric Obesity Population

The development of the paediatric obesity population in Simcyp^®^ software Version 21 was adapted from the paediatric population file with the weight and height parameters for obese children which were modified and derived based on the defined obese growth charts published by the CDC in 2000 [13], the WHO in 2006 and 2007 [12], as well as Gerhart et al. (2022) [11]. The weight and height changes with age altered all other parameters, which include haematocrit, serum albumin, and AGP, as shown in Equations (S5)–(S8) of the Supplementary Materials. In addition, age is also related to blood flow and tissue-water composition. As for the GFR, the changes are influenced by BSA as per Equation (S9) (Supplementary Materials), which is directly related to weight and height changes. Additionally, weight and height have a direct relationship with organ size. All the equations to address the relationships were defaulted in Simcyp^®^ except for age, weight, and height (Equations (S1)–(S4) (Supplementary Materials)).

For validation of the paediatric obesity population file, verification focused on the relationship between age and BMI, weight, height, protein binding, and GFR because of the availability of published data for obese children. As reported in the Results section of the Supplementary Materials, the predicted parameter distributions for all six parameters aligned with the data reported by various publications involving paediatric obesity. Haematocrit, serum albumin, and AGP showed no specific trend with age, which concurs with other simulations of paediatric obesity populations and agrees with various publications that report data for paediatrics with and without obesity [11,31,44,96].

For GFR, the BSA-adjusted GFR-to-age plot showed no specific trend, as the weight and height were annulled when plotting the chart (Figure S8). However, an increasing trend can be seen when plotting the absolute GFR-to-age graph, which is in line with the increased kidney volume trend [11]. Additionally, reports showed no statistically significant difference in GFR between obese and normal children, which is in agreement with Equation (S9) (Supplementary Materials) that used BSA to simulate the absolute GFR trend with age [10,16,45].

Information on changes in metabolic enzymes and transporter abundance, which play an essential role in metabolism and elimination, is still limited for paediatric obesity populations; thus, we choose to keep the default trend for the paediatric population. Information on the metabolic enzyme changes was only reported in obese adults, for example, the CYP3A4 activity was reduced by 40% in obese adults [8]. A study by van Rongen et al. [9] reported higher clearance for midazolam, a substrate of CYP3A4, in obese adolescents compared to obese adults, which is the opposite of what was noted in obese adults and may be due to comorbidities and other factors.

### 4.2. Step 2: Validation of Paediatric Population with Metformin and Ceftazidime

The paediatric obesity population was further validated with metformin and ceftazidime. For the metformin, the compound file available in Simcyp^®^ was used with minor adjustments to fa and V_ss_. As standard practice in verifying modified and newly developed compound files, the adapted metformin file was verified in healthy and obese adults as well as obese and non-obese paediatric populations [21,89,97,98,99]. All simulated pharmacokinetic parameters and plasma concentration profiles generated with metformin were within the acceptance criteria and hence demonstrated validation.

Subsequently, ceftazidime has been previously developed/validated by Zhou et al. (2019) [59,60,61,62] and was used without any adaptation to verify the paediatric obesity population. The reported steady-state plasma concentration data of ceftazidime in obese children used for validation were sparse with a summary of the median, minimum, and maximum dosing information [58]. Acceptance of the result was based on VPC alone, as only the population pharmacokinetic parameter estimates were reported. Nonetheless, the median C_max_ and clearance estimates in the population pharmacokinetic study are comparable with this study (5.40 L/h vs. 6.25 L/h), which complements the VPC result on validating the paediatric obesity population file [58].

### 4.3. Step 3: Validation of the Amlodipine Model

The amlodipine compound file that was developed based on compilations of information and parameter optimisation from several publications was verified in four populations, including non-obese adults, obese adults, non-obese paediatrics, and obese paediatrics [63,64,65]. The simulated plasma concentrations and pharmacokinetic parameters for non-obese adults and obese adults met the acceptance criteria for the VPC and 2-fold comparison with observed data except for 3 out of 54 comparisons.

Firstly, the plasma profiles were compared between the simulated single-dose intravenous study in healthy adults and the intravenous plasma profile published by Faulkner et al. (1986) [74], with only 3 out of 16 points at the distal region of the elimination phase not within the 5th and 95th percentiles. Nevertheless, the simulated AUC_inf_ for intravenous was within the 2-fold ratio compared to the reported value.

Secondly, the plasma concentration profiles were compared between the simulated 15 mg daily dose for 14 days and the observed profile reported by Faulkner et al. (1986) [74], in which the three last points were not within the 5th and 95th percentiles of the simulated profiles. However, all the simulated pharmacokinetic parameters for the same study are within 2-fold of the reported parameters. Further, simulated amlodipine plasma concentration profiles at steady state were stable between the low and high doses due to the simulated plasma profile being overpredicted when administered with 15 mg per day (Figure 5C) and underpredicted when dosed with 5 mg daily (Figure 5B).

Thirdly, the simulated AUC_infss_ of 5 mg daily for 14 days was not within 2-fold compared to the reported AUC_infss_ by Bainbridge et al. (1993) [75]. However, all other parameters were within 2-fold, and the published plasma concentration was within the 5th and 95th percentiles of the simulated profile. Additionally, the AUC_infss_ is seldom utilised for pharmacokinetic parameter comparisons, especially within regulatory contexts, due to its reliability, mainly when the percentage difference between AUC_inf_ and AUC_0-t_ is more than 20%, which is exemplified in this case, where the difference was undisclosed and exceeded 20% for the reported and simulated results, respectively [100]. In addition, the number of samples utilised for AUC_inf_ extrapolation between observed and simulated results (6 vs. 24) potentially overestimates the parameters of one over the other [101].

Acceptance of the simulation in paediatric populations with and without obesity was based on the VPC alone because, based on our search, only sparse data on amlodipine concentrations were available in paediatric populations [72,73]. Simulated plasma concentrations that mimicked the study design and dosing range of both studies fell in the middle of the reported plasma concentration data precisely when simulations were performed based on the mean and median doses [72,73]. Additionally, the proportion of obese children in the simulated population was comparable to that reported by Flynn et al. (2006) [73] (50.73–50.86% versus 43.2%). The results validated the amlodipine and paediatric obesity population files, which were then used to explore and optimise amlodipine dose in obese children.

### 4.4. Step 4: Impact of Obesity on Amlodipine Pharmacokinetics and Dose Optimisation in Obese Paediatric Population

#### 4.4.1. Influence of Obesity on Amlodipine Pharmacokinetics

The trend of plasma concentration profiles, C_max_, and AUC decreased as the age group increased when the dose was fixed (Figure 8 and Figure 9). Nevertheless, when the dose was fixed at 2.5 mg, the dose amount per kilogram of body weight was higher in the lower age groups compared to the higher age groups in obese and non-obese populations, with a 0.07 to 0.26 mg/kg dose in the 2 to 6 years old group, 0.03 to 0.18 mg/kg in the 6.01 to 12 years old group, and 0.02 to 0.10 mg/kg in the 12.01 to 18 years old group. The same pattern can be seen in other studies where younger patients required higher doses per kilogram of body weight than older children [73,92,102].

Amlodipine is metabolised in the liver, particularly by CYP3A4 [103]. Thus, the change in hepatic-to-body-size ratio and expression of CYP3A4 enzymes from infancy to adolescence was speculated to be the reason [73,103,104]. Additionally, the weight-normalised clearance for the simulation showed an inverse trend with age, which aligns with the theory. Furthermore, the pattern is similar to carbamazepine, where the CYP3A4 enzyme influences the clearance of carbamazepine significantly, and a few studies showed that a higher weight-adjusted dose is required for children to achieve the same effect [105,106,107].

Significantly lower plasma concentration, C_max_, and AUC were noted in the obese compared to the non-obese paediatric populations within the same age group when a fixed dose was administered (Figure 9). The volume of distribution for obese cases is higher than for non-obese children, explaining the requirement for higher doses in the obese population (Figure S9) [108]. Generally, the volume of distribution has an inverse relationship with plasma concentration, where a high volume of distribution of the drug occurs in the tissues [108].

Another factor is clearance, which is higher in obese children than non-obese children (Figure S9). Since amlodipine is cleared through the liver, CYP3A4, liver size, and blood flow to the liver may be the factors that lead to higher clearance in obese children [94]. Simulation data presented that liver weight and blood flow to the liver are higher in the obese than in non-obese paediatric populations. In contrast, the difference in CYP3A4 abundance between obese and non-obese children is subtle. Furthermore, the discovery is consistent with numerous studies involving midazolam, another CYP3A4 substrate, which have observed reduced plasma concentrations in obese compared to non-obese children [9,109,110].

In addition, the result agrees with the finding reported by Hanafy et al. (2009) [26] that obese children demonstrate a considerably reduced response to amlodipine in lowering systolic blood pressure and response rate compared to non-obese children. Based on animal studies, plausible explanations speculated by Hanafy et al. (2009) include the downregulation of the L-type calcium channel receptor due to the inflammatory conditions in hypertension, exacerbated further by the obese state [111,112]. Additionally, the same can be seen in verapamil, another CCB, where the effect of verapamil is reduced in obese adult patients, and another study involving rheumatoid arthritis patients showed that despite the increase in verapamil concentration, the response is diminished, aligning with the theory that increased in inflammation mediators possibly reduced the L-calcium channel receptor [113,114].

#### 4.4.2. Dose Adjustment in Paediatric Obesity

From a pharmacokinetic perspective, this study shows that a weight-based dose is suitable for paediatric obesity to achieve the same concentration range as in non-obese children, specifically for those aged 2 to 12 years old (Figure 10). Considering obese paediatrics are heavier, a larger dose was administered based on the weight-based dosing scheme, compensating for the higher clearance and volume of distribution in obese children and leading to the same exposure as in the non-obese population. The finding aligns with the recommendation to use weight-based dosing for the same age group by the BNFc and the European Society for Hypertension in their 2016 clinical practice guideline [25,79]. Additionally, oral solution and suspension availability in the market made weight-based dosing straightforward for children. Regarding the choice of body weight, the use of TBW in this simulation was appropriate for amlodipine, given its lipophilic nature [115].

On the other hand, with a weight-based dosing regimen, a significant percentage of obese children are expected to reach the maximum daily dose. Therefore, close monitoring of possible side effects related to amlodipine, such as oedema, palpitations, abdominal pain, flushing, dizziness, and others, is essential since a higher dose is likely to result in amlodipine concentrations above the suggested therapeutic upper limit (57.2 ng/mL) and toxicity range (67 ng/mL) [80,92,93].

Based on the amlodipine product insert and clinical practice guidelines by the American Academy of Pediatrics in 2017, for children 6 years old and older, a fixed-dose regimen starting at 2.5 mg daily with a maximum of 5 mg and 10 mg per day, respectively, is recommended [24,80]. Simulations demonstrated that a 1.25- to 1.5-fold higher dose in obese children is required to achieve the same amlodipine concentration as in non-obese paediatrics (Figure 10).

Therefore, a higher initial dose of 3.75 mg daily may be considered in obese children across the 6 to 18 years age group to assist in maintaining blood pressure instantaneously, mainly when physicians opt for the fixed-dose regimens. Although the result showed a significant difference in C_max_ between obese and non-obese children for the starting dose in the 16 to 17 years old age group, it may not be clinically significant as the amlodipine concentrations were still within the therapeutic limit.

Following treatment initiation, systolic and diastolic blood pressure readings and side effects are fundamental factors that drive the dose adjustment, which is practically made after 1 to 2 weeks of the initial dose [24,79]. Considering therapeutic drug monitoring for any antihypertensive agent is uncommon unless to evaluate medication compliance, the difference in amlodipine plasma concentrations between obese and non-obese children will have minimal influence as the deciding factor in making dose adjustments, specifically after the treatment has started [116].

Amlodipine is available in both solid and liquid dosage forms. Thus, the fixed-dose regimen may be suitable for specific age groups, such as children above 13 years old, as a study showed approximately 30% of children aged 13 to 18 years old favoured tablets rather than liquid formulations (18.3%) [117].

Based on the pharmacokinetic study of amlodipine in children by Flynn et al. (2006) [73], amlodipine concentrations of 1–57.2 ng/mL demonstrated no serious adverse events. Thus, this study showed that for children 6 years old and above, a fixed-dose regimen is expected to maintain the amlodipine concentrations within the therapeutic range and reduce the harm that potential adverse events may cause with higher doses. Since significant differences in amlodipine concentration between obese and non-obese children were noted at 9 years old with weight-based doses, the fixed dose can be considered at 9 years old and above. Nevertheless, any dose below 0.35 mg/kg daily is unlikely to cause side effects as less than 20% of the simulated C_max_ surpasses the maximum therapeutic range.

## 5. Conclusions

For the first time, mechanistic pharmacokinetic modelling is implemented in this study by establishing a virtual paediatric obesity population as a pragmatic approach to address the impact of obesity on drug pharmacokinetics.

Our findings highlight that a suitable dose adjustment is required to achieve the same amlodipine plasma concentration as in non-obese children. The physiological alteration in obese paediatrics led to a significant difference in amlodipine C_max_ and AUC when administered as a fixed-dose regimen compared to non-obese children. Thus, when opting for a fixed-dose regimen, a 1.25- to 1.5-fold higher dose is needed in obese children to achieve a comparable amlodipine plasma concentration to non-obese children.

This study highlights the potential of PBPK modelling and its application to addressing personalised dosing in the obese paediatric population. Further improvements can be made with the virtual paediatric obesity population group by refining the physiological information, such as changes in the metabolism enzymes specific to obese children as they evolve with age, and the findings from this study will inform medicine optimisation approaches in future studies.

## Figures and Tables

**Table 1:** Metformin compound parameters used for validation studies.

Parameters	Values	Notes
Physical chemistry and blood binding		
Compound type	Monoprotic base	
Molecular weight (g/mol)	129.16	
Log P	−1.43	
pKa 1	11.8	
fu	1	
B/P	1	
Absorption		
Model	1st order	
fa	0.45	Fitted based on reported values [47,48].
ka (1/h)	0.27	
Lag time (h)	0.29	
Distribution		
Model	Full PBPK	
V_ss_(L/kg)	1.0172	Predicted using Rodgers and Rowland method [49,50].
Kp scalar	1	Fitted based on observed profiles [47,52].
Elimination (enzyme kinetics)		
Pathway 1	CYP3A4	
CL_int_ (µL/min/pmol—isoform)	0.334	
fu_mic_	1	
Renal clearance (L/h)	32.3	
Drug transport		
Pathway 1 (Liver)	SLC22A1 (OCT1)	
CL_int,T_ (µL/min/million—cells)	0.316	
fu_inc_	1	
RAF/REF	1.84	
CL_PD_ (mL/min/million hepatocytes)	0.0000588	
Pathway 2 (Kidney)	SLC22A2 (OCT2)	
CL_int,T_ (µL/min/million—cells)	14.21	
J_max_	21084	
Km (µmol)	1483	
Pathway 3 (Kidney)	SLC47As (MATEs)	
CL_int,T_ (µL/min/million—cells)	16.64	
RAF/REF	0.128	
J_OCT2_ (pmol/min/millivolt/million cells)	1.155	

Log P, partition coefficient; B/P, blood-to-plasma ratio; fu, unbound fraction; V_ss_, steady-state volume of distribution; Kp scalar, tissue partition coefficient; ka, absorption rate constant; fa, extent of absorption; CL_int_, in vitro intrinsic clearance; fu_mic_, fraction of unbound drug in the in vitro microsomal incubation; CL_int,T_, in vitro transporter-mediated intrinsic clearance; fu_inc_, fraction of unbound drug in the in vitro microsomal incubation; RAF/REF, relative activity factor or relative expression factor; CL_PD_, passive diffusion clearance; J_max_, in vitro maximum rate of transporter-mediated efflux or uptake; Km: Michaelis constant; J_OCT2_, in vitro OCT2 flux per unit of electrochemical gradient.

**Table 2:** Datasets of Metformin and Ceftazidime used for validation of paediatric obesity population.

Reference	Subjects	Age (Years)	Dose Regimen	PK Sampling
Metformin				
Healthy adult subjects				
[47]	4 males	30–36	Single-dose 500 mg—fed state (oral)	Up to 24 h post-dose
[52]	14 (7 males, 8 females)	37.0 ± 7.7	Single-dose 500 mg—fed state (oral)	Up to 24 h post-dose
[53]	15 (9 males, 7 females)	19–40	1000 mg twice daily (oral)	Up to 24 h post-dose at steady state
Obese adults				
[54]	16 (3 males, 13 females) BMI: 40.5 ± 6.9	43.5 ± 11.7	Single-dose 1000 mg—fast state (oral)	Up to 24 h post-dose
Paediatric subjects				
[55]	4 females	9	850 mg once daily—fed state (oral)	Up to 24 h post-dose at steady state
Paediatric obesity subjects				
[56]	22 (6 males, 16 females) (5 overweight, 17 obese)	11.1–17.5	1000 mg twice daily (oral)	Up to 8 h post-dose at steady state
[57]	28 obese paediatrics	7.7–13.5	1000 mg twice daily (oral)	Up to 12 h post-dose at steady state
Ceftazidime				
[58]	29 (17 males, 12 females) (82.80% obese)	2.3–20.6	Median: 33.8 mg/kg/dose, Lowest–highest: 16.5–92.9 mg/kg/dose, maximum dose: 2 g/dose (intravenous every 8 h)	Post-dose sparse sampling after at least 8 doses

(n–n), age range; mean ± SD; BMI, body mass index.

**Table 3:** Ceftazidime compound parameters used for validation and simulation.

Parameters	Values	Notes
Physical chemistry and blood binding		
Compound type	Diprotic acid	
Molecular weight (g/mol)	546.58	
Log P	−3.75	
pKa (1/2)	2.43, 2.89	
fu	0.85	
B/P	0.55	
Distribution (full PBPK)		
V_ss_(L/kg)	0.22	Predicted using Rodgers and Rowland method [49,50].
Kp scalar	1.03	
Elimination		
Renal clearance (L/h)	6	
Additional systemic clearance (L/h)	0.9	

Log P, partition coefficient; B/P, blood-to-plasma ratio; fu, unbound fraction; V_ss_, steady-state volume of distribution; Kp scalar, tissue partition coefficient.

**Table 4:** Amlodipine compound parameters used in validation and simulation.

Parameters	Values	Notes
Physical chemistry and blood binding		
Compound type	Diprotic base	
Molecular weight (g/mol)	408.88	
Log P	3.43	[63]
pKa 1	9.40	[63]
pKa 2	1.90	[63]
fu	0.07	[63]
B/P	0.71	Predicted by Simcyp^®^.
Absorption		
Model	ADAM	Permeability limited model.
f_uGut_	0.20	[65]
P_eff_ in man (10^−4^ cm/s)	0.289	Predicted by Simcyp^®^ from PSA/HBD.
PSA (Å^2^)	105.50	[63]
HBD	3.00	[63]
Distribution		
Model	Full PBPK	
V_ss_ (L/kg)	36.12	Predicted using Rodgers and Rowland method [49,50].
Kp scalar	22.70	An estimate based on observed data [67].
Elimination (enzyme kinetics)		
HLM CL_int_ by CYP3A4 (µL/min/mg—microsomal)	42.40	[68]
Additional HIMel CLint (µL/min/mg—microsomal)	22.00	[69]
Renal clearance (L/h)	5.77	[64]

Log P, partition coefficient; B/P, blood-to-plasma ratio; fu, unbound fraction; ADAM, advance dissolution, absorption, and metabolism; P_eff_, human jejunum effective permeability; PSA, polar surface area; HBD, number of hydrogen bond donors; f_uGut_, unbound fraction of drug in enterocytes; V_ss_, steady-state volume of distribution; Kp scalar, tissue partition coefficient; HLM CL_int_, human liver microsomes in vitro intrinsic clearance; HIMel CL_int_, human intestinal microsomes in vitro intrinsic clearance.

**Table 6:** Observed versus predicted pharmacokinetic parameters for metformin.

Study	Dosing	PK Parameters	Observed	Predicted	Predicted/ Observed
Healthy adults					
[47]	500 mg once	C_max_ (mcg/L)	1.02 ± 0.34	0.78 ± 0.28	0.77
		AUC_0–24_ (h.mcg/mL)	6.71 ± 1.82	6.70 ± 2.16	1.00
		T_max_ (h)	2.20 ± 0.30	2.62 ± 0.70	1.19
[52]	500 mg once	C_max_ (ng/mL)	741.00 ± 175.00	782.22 ± 277.48	1.06
		AUC_0–24_ (h.ng/mL)	5330.00 ± 1400.00	6696.68 ± 2158.24	1.25
		T_max_ (h)	3.50 ± 0.70	2.62 ± 0.70	0.75
[53]	1000 mg twice daily	C_maxss_ (ng/mL)	1321.00 ± 234.00	1898.97 ± 630.13	1.44
		AUC_0–24ss_ (h.ng/mL)	20,544.00 ± 4445.00	28,806.57 ± 9843.03	1.40
		T_max_ (h)	3.00 (1.50–6.00)	2.32 (1.35–3.45)	0.77
Obese adults					
[54]	1000 mg once	C_max_ (mcg/mL)	1.80 ± 0.61	1.37 ± 0.49	0.76
		AUC_0–24_ (h.mcg/mL)	11.10 ± 3.60	11.89 ± 4.15	1.07
		T_max_ (h)	3.00 (1.5–3.0)	2.75 (1.60–4.90)	1.16
Paediatric subjects					
[55]	850 mg once daily	C_maxss_ (mg/L)	3.10 ± 0.30	3.40 ± 1.12	1.10
		AUC_0–12ss_ (h.mg/L)	21.20 ± 1.50	24.18 ± 9.40	1.14
		T_max_ (h)	2.40 ± 0.20	2.78 ± 0.56	1.16
Paediatric obesity subjects					
[57]	1000 mg twice daily	C_maxss_ (mg/L)	2.80 ± 0.98	2.44 ± 1.06	0.87
		AUC_0–12ss_ (h.mg/L)	14.30 ± 5.00	18.64 ± 9.87	1.30
		CL/F (mL/min)	1007.00 ± 326.00	1108.83 ± 524.17	1.10
[56]	1000 mg twice daily	C_maxss_ (mg/L)	1.80 (0.79–3.45)	1.64 (0.68–4.95)	0.91
		AUC_0–8ss_ (h.mg/L)	10.06 (4.78–18.66)	10.13 (3.59–33.83)	1.01
		T_max_ (h)	2.00 (1.00–4.00)	2.50 (1.40–3.55)	1.25

Mean ± SD; median (range).

**Table 7:** Observed and predicted amlodipine pharmacokinetic parameters in adults.

Study	Dosing	PK Parameters	Observed	Predicted	Predicted/ Observed
Adult populations					
[74]	Single-dose 10 mg IV	AUC_0-inf_ (h.ng/mL)	371.00 ± 69.00	668.60 ± 197.38	1.80
	Single-dose 10 mg oral	C_max_ (ng/mL)	5.90 ± 1.20	6.10 ± 2.45	1.03
		AUC_0-inf_ (h.ng/mL)	238.00 ± 53.00	373.21 ± 132.47	1.57
		T_max_ (h)	7.60 ± 1.80	5.06 ± 0.93	0.67
	15 mg oral daily for 14 days	Day 1: C_max_ (ng/mL)	6.90 ± 2.60	6.92 ± 1.60	1.00
		Day 1: C_min_ (ng/mL)	3.30 ± 1.20	3.36 ± 0.90	1.02
		Day 1: T_max_ (h)	8.90 ± 3.70	5.50 ± 0.79	0.62
		Day 14: C_max_ (ng/mL)	18.10 ± 7.10	23.55 ± 7.09	1.30
		Day 14: C_min_ (ng/mL)	11.80 ± 5.30	8.17 ± 3.93	0.69
		Day 14: T_max_ (h)	8.70 ± 1.90	4.92 ± 0.60	0.57
[67]	Single-dose 2.5 mg	C_max_ (ng/mL)	1.20	1.52 ± 0.61	1.27
		AUC_0–72_ (h.ng/mL)	41.00	46.51 ± 17.13	1.13
		T_max_ (h)	5.40	5.06 ± 0.93	0.94
	Single-dose 5 mg	C_max_ (ng/mL)	2.66	3.05 ± 1.23	1.15
		AUC_0–72_ (h.ng/mL)	94.00	93.10 ± 34.30	0.99
		T_max_ (h)	6.30	5.06 ± 0.93	0.80
	Single-dose 10 mg	C_max_ (ng/mL)	5.49	6.10 ± 2.45	1.11
		AUC_0–72_ (h.ng/mL)	200.00	186.52 ± 68.78	0.93
		T_max_ (h)	6.4	5.06 ± 0.93	0.79
[66]	2.5 mg once daily	C_maxss_ (ng/mL)	4.20 ± 1.10	3.90 ± 1.32	0.93
		AUC_0–24ss_ (h.ng/mL)	81.00 ± 22.00	77.49 ± 26.36	0.96
		T_maxss_ (h)	7.00 ± 2.00	4.54 ± 0.72	0.65
[75]	Single-dose 5 mg	C_max_ (ng/mL)	3.50 ± 0.80	3.05 ± 1.23	0.87
		AUC_0-inf_ (h.ng/mL)	169.00 ± 53.00	145.60 ± 55.19	0.86
		T_max_ (h)	6.80 ± 1.80	5.06 ± 0.93	0.74
	5 mg once daily for 14 days	C_maxss_ (ng/mL)	10.50 ± 4.40	8.51 ± 2.82	0.81
		AUC_0-infss_ (h.ng/mL)	214.00 ± 78.00	885.10 ± 462.87	4.14
		T_maxss_ (h)	7.00 ± 1.00	4.53 ± 0.71	0.65
[76]	Single-dose 10 mg	C_max_ (ng/mL)	4.30 ± 0.90	6.10 ± 2.45	1.42
		AUC_0–72_ (h.ng/mL)	163.00	186.52 ± 68.78	1.14
		T_max_ (h) ^a^	7.00 (5.00–12.00)	4.98 (2.85–7.40)	0.71
[77]	Single-dose 5 mg	C_max_ (ng/mL)	2.40 ± 0.20	3.05 ± 1.23	1.27
		AUC_0–24_ (h.ng/mL)	42.00 ± 3.40	49.54 ± 18.60	1.18
		T_max_ (h)	6.90 ± 0.60	5.06 ± 0.93	0.73
	5 mg once daily for 8 weeks	C_maxss_ (ng/mL)	8.10 ± 0.60	9.52 ± 3.25	1.18
		AUC_0–24ss_ (h.ng/mL)	162.90 ± 13.80	194.63 ± 71.84	1.20
		AUC_0–240ss_ (h.ng/mL)	594.50 ± 58.20	949.43 ± 519.01	1.60
		T_maxss_ (h)	6.40 ± 0.60	4.48 ± 0.69	0.70
Obese adult					
[78]	5 mg daily 10 mg daily	C_maxss_ (ng/mL)	24.88 ± 13.87	14.75 ± 6.68	0.59
		AUC_0–72ss_ (h.ng/mL)	1176.38 ± 704.86	794.80 ± 383.20	0.68
		AUC_0-infss_ (h.ng/mL)	2387.34 ± 1705.50	2270.93 ± 1474.58	0.95
		T_max_ (h)	5.33 ± 1.97	5.01 ± 0.76	0.94

^a^ Median (range); IV, intravenous.

## Data Availability

The data presented in this study are available in the article and Supplementary Materials.

## Supplementary Materials

The following supporting information can be downloaded at: https://www.mdpi.com/article/10.3390/pharmaceutics16040489/s1, Supplementary results; Supplementary figures, Figure S1: Simulated BMI-for-age curves for paediatric obesity from 2 to 18 years old for males (A) and females (B). Gerhart et al. (2022) generated the paediatric obesity BMI-for-age curve at the 95th percentile based on the National Health and Nutrition Examination Survey (NHANES) pooled data from 1999 to 2016. The CDC’s 2000 BMI-for-age curve is at the 95th percentile, which defines the cut-off curve for obesity in paediatrics (CDC, 2017). The WHO, 2006 BMI-for-age curve is at 3 SD from the median for 2 to 5 years old, while the WHO, 2007 (WHO, 2021) one is at 2 SD from the median for 6 to 18 years old (WHO, 2021); Figure S2. Simulated height-for-age curve for paediatric obesity from 2 to 18 years old for males (A) and females (B). Gerhart et al. (2022) generated the central tendency of paediatric obesity’s height-for-age curve based on the National Health and Nutrition Examination Survey (NHANES) pooled data from 1999 to 2016; Figure S3. Simulated weight-for-height curves for paediatric obesity from 2 to 18 years old for males (A) and females (B). Gerhart et al. (2022) generated the central tendency of paediatric obesity’s weight-for-age curve based on the NHANES pooled data from 1999 to 2016; Figure S4. Simulated weight-for-height curves for paediatric obesity from 2 to 5 years old for males (A) and females (B). The CDC, 2000 (A) weight-for-height curve is at the 97th percentile (CDC, 2017). The CDC, 2000 (B) weight-for-height curve is at the 95th percentile, which defines the cut-off curve for obesity in paediatrics (CDC, 2017). The WHO, 2006 weight-for-age curve is at 3 SD from the median for 2 to 5 years old, which defines the cut-off curve for obesity in paediatrics (WHO, 2021); Figure S5. Simulated haematocrit-to-age relationship for paediatric obesity from 2 to 8 years old (grey circle). Gerhart et al., 2022 reported individual haematocrit data for obese children from combined clinical trials data represented by the red circles (Gerhart et al., 2022). Jeong et al., 2021 (A) represented data for girls (Jeong et al., 2021). Jeong et al., 2021 (B) represented data for boys (Jeong et al., 2021). Belo et al., 2014 (A) and Belo et al., 2014 (B) represented data for girls and boys, respectively (Belo et al., 2014). Elhag et al., 2018 (A) represented data for girls (Elhag et al., 2018). Elhag et al., 2018 (B) represented data for boys (Elhag et al., 2018). The horizontal lines shows the age range reported for each published study. The coloured circles with the vertical lines are different for each study; Kilic et al., 2016, median with range; Panichsillaphakit et al., 2021, median with interquartile range; Oni et al., 2021, mean with 95% confidence interval; Jeong et al., 2021 mean with standard deviation (SD); Belo et al., 2014, mean with SD; Cacciari et al., 1988, mean with SD; Elhag et al., 2018, mean with SD; Figure S6. Predicted serum-albumin-to-age relationship for paediatric obesity from 2 to 18 years old (grey circle). Gerhart et al., 2022 (A) reported individual serum albumin data for children with obesity from combined clinical trials data, shown by red circles (Gerhart et al., 2022). Gerhart et al., 2022 (B) reported individual data for paediatric obesity from the Paediatric Trial Network (PTN) data repository (Gerhart et al., 2022). The horizontal lines show the age range reported for each published study. The coloured squares with vertical lines represent the mean with SD; Figure S7. Predicted AGP-to-age relationship for paediatric obesity from 2 to 18 years old (grey circle). Sobieska et al., 2013 (A) represented data for boys aged 12 to 14 (Sobieska et al., 2013). Sobieska et al., 2013 (B) represented data for girls aged 12 to 14 (Sobieska et al., 2013). Sobieska et al., 2013 (C) represented data for boys 15 to 18 years old (Sobieska et al., 2013). Sobieska et al., 2013 (D) represented data for girls 15 to 18 years old (Sobieska et al., 2013). The horizontal lines show the age range reported for each published study. The coloured squares with vertical lines represent the mean with SD for Gerhart et al. (2022) and Sobieska et al. (2013). The coloured squares with vertical lines represent the median with range for Gibson et al. (2014) and Ferrari et al. (2015); Figure S8. Absolute GFR (mL/min)-to-age relationship for paediatric obesity from 8 to 9 years old (A) and BSA-adjusted GFR (mL/min/1.73 m^2^)-to-age correlation for paediatric obesity from 8 to 9 years old (B). Grey circles are the predicted value. The horizontal lines show the age range reported for each published study. The coloured squares with vertical lines represent the mean with SD; Figure S9: Comparison of predicted clearance (A) and volume of distribution (B) at steady state for healthy and obese paediatric doses; Figure S10: Predicted C_max_ versus daily doses for age group 2 to 6 years old; Figure S11: Predicted C_max_ versus daily doses for age group 6.01 to 12 years old; Figure S12: Predicted C_max_ versus daily doses for age group 12.01 to 18 years old; Figure S13: Summary of pharmacokinetic parameters at steady state in healthy paediatrics from 2 to 18 years old; Figure S14: Summary of pharmacokinetic parameters at steady state in paediatric obesity from 2 to 18 years old administered with weight-based dose; Figure S15: Summary of pharmacokinetic parameters at steady state in paediatric obesity from 2 to 18 years old administered with fixed dose. Table S1. Summarised results from literature search for haematocrit values in paediatric obesity; Table S2. Summarised results from literature search for serum albumin values in paediatric obesity; Table S3. Summarised results from literature search for AGP values in paediatric obesity; Table S4. Summarised results from literature search for GFR values in paediatric obesity.
