# Supplementary material for: The Impact of Paediatric Obesity on Drug Pharmacokinetics: A Virtual Clinical Trials Case Study with Amlodipine
Source: Pharmaceutics. 2024 Apr 2;16(4):489. doi: 10.3390/pharmaceutics16040489 (PMC11053426; doi:10.3390/pharmaceutics16040489)
Supplement: Supplementary file 1 [file pharmaceutics-16-00489-s001.zip › pharmaceutics-2866575-supplementary.pdf]

A paediatric obesity virtual clinical trial: a case study with amlodipine

Khairulanwar Burhanuddin[1], Afzal Mohammed[1], Raj K. S. Badhan[1]
[1] Medicines Optimisation Research Group, Aston Pharmacy School, College of Health and Life Sciences, Aston University, Birmingham, B4 7ET, United Kingdom.

Supplementary materials

1. Supplementary results

1.1. Step 1: Development of the paediatric obesity population

Age, weight and height relationship

Polynomial mathematical correlations for gender-specific of height-age and weight-age for the population are described in Equations (1), (2), (3) and (4) for 2 – 18 years old:

$$\text{Male height (cm)} = 61.463912 + (0.65513619 \times \text{age}^2) - (0.12642816 \times \text{age}^{2.5}) + (17.3579 \times \text{age}^{0.5}) \quad (1)$$

$$\text{Female height (cm)} = 64.092976 + (10.720395 \times \text{age}) - (0.29561165 \times \text{age}^2) \quad (2)$$

$$\text{Male weight (kg)} = 3.2737 + (0.5346053 \times \text{age}^2) - (0.015322545 \times \text{age}^3) \quad (3)$$

$$\text{Female weight (kg)} = 11.809275 + (0.64670616 \times \text{age}^2) - (0.022469269 \times \text{age}^3) \quad (4)$$

The WHO and CDC graphs, which are the cut-off lines for children's obesity overlayed at the lower part of the individual simulated BMI-for-age graph (2 – 18 years old) for males and females (S1), indicated that the predictions represent the BMI for the obese children population (WHO, 2021, CDC, 2017). For the mean BMI-for-age graph of paediatric obesity, the reference curves by Gerhart et al. (2022) cover the predicted chart, thus validating the simulated BMI-to-age chart.

Additionally, a similar pattern can be seen for the height-for-age curves (2 – 18 years old) for males and females (Figure S2), as well as the weight-for-height curves (2 – 18 Years old) for males and females (Figure S3). The reference graphs of mean height-for-age and mean weight-for-height of the paediatric obesity population published by Gerhart et al. (2022) fit in the middle of the simulated graphs. Thus, it further verified the predicted weight and height of the paediatric obesity population.

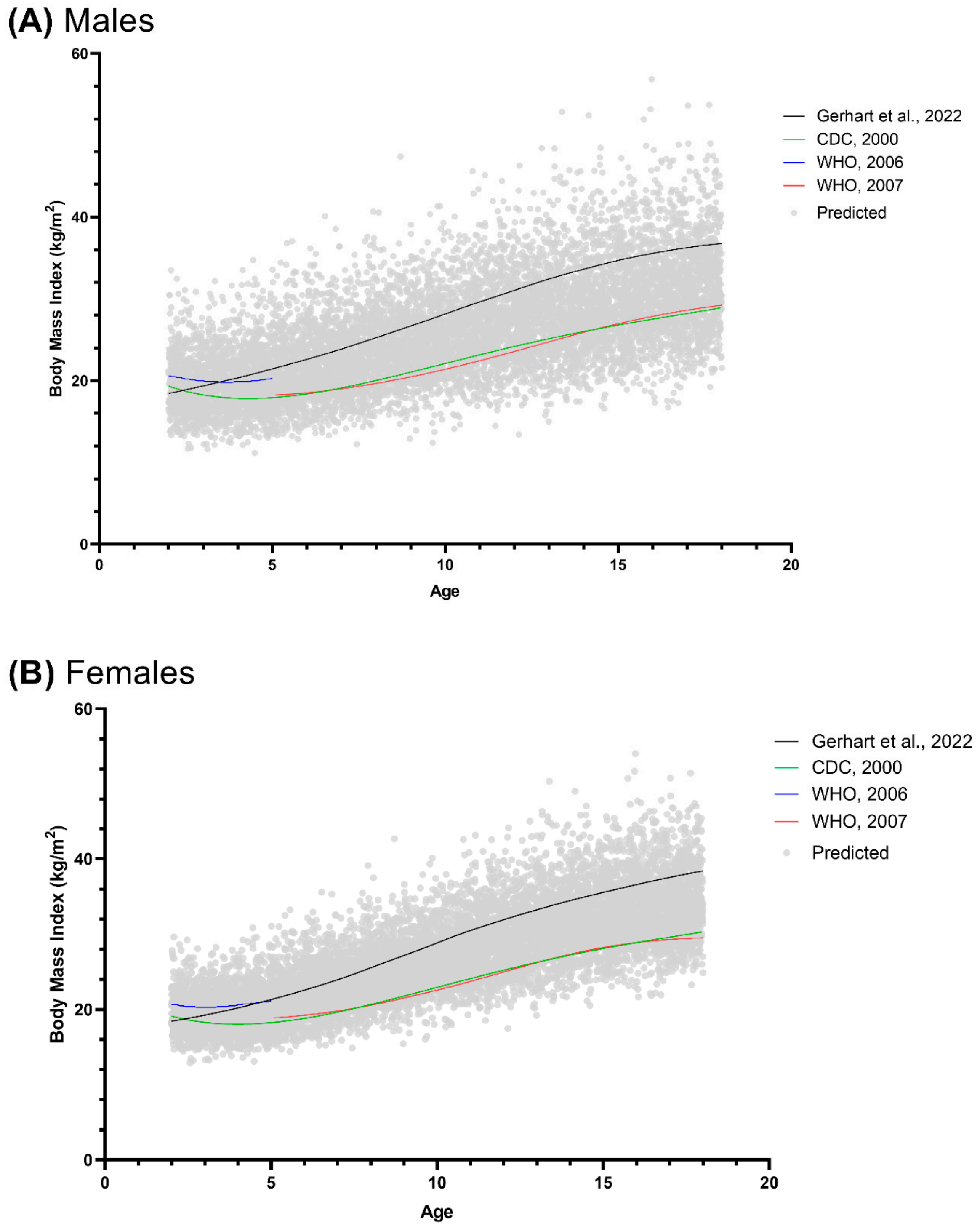

Figure S1. Simulated BMI-for-age curves for paediatric obesity from 2 to 18 years old for males (A) and females (B). Gerhart et al. (2022) generated the paediatric obesity BMI-for-age curve at the 95th percentile based on the National Health and Nutrition Examination Survey (NHANES) pooled data from 1999 to 2016. The CDC's 2000 BMI-for-age curve is at the 95th percentile, which defines the cut-off curve for obesity in paediatrics (CDC, 2017). The WHO, 2006 BMI-for-age curve is at 3 SD from the median for 2 to 5 years old, while the WHO, 2007 (WHO, 2021) is at 2 SD from the median of the BMI-for-age curve for 6 to 18 years old (WHO, 2021).

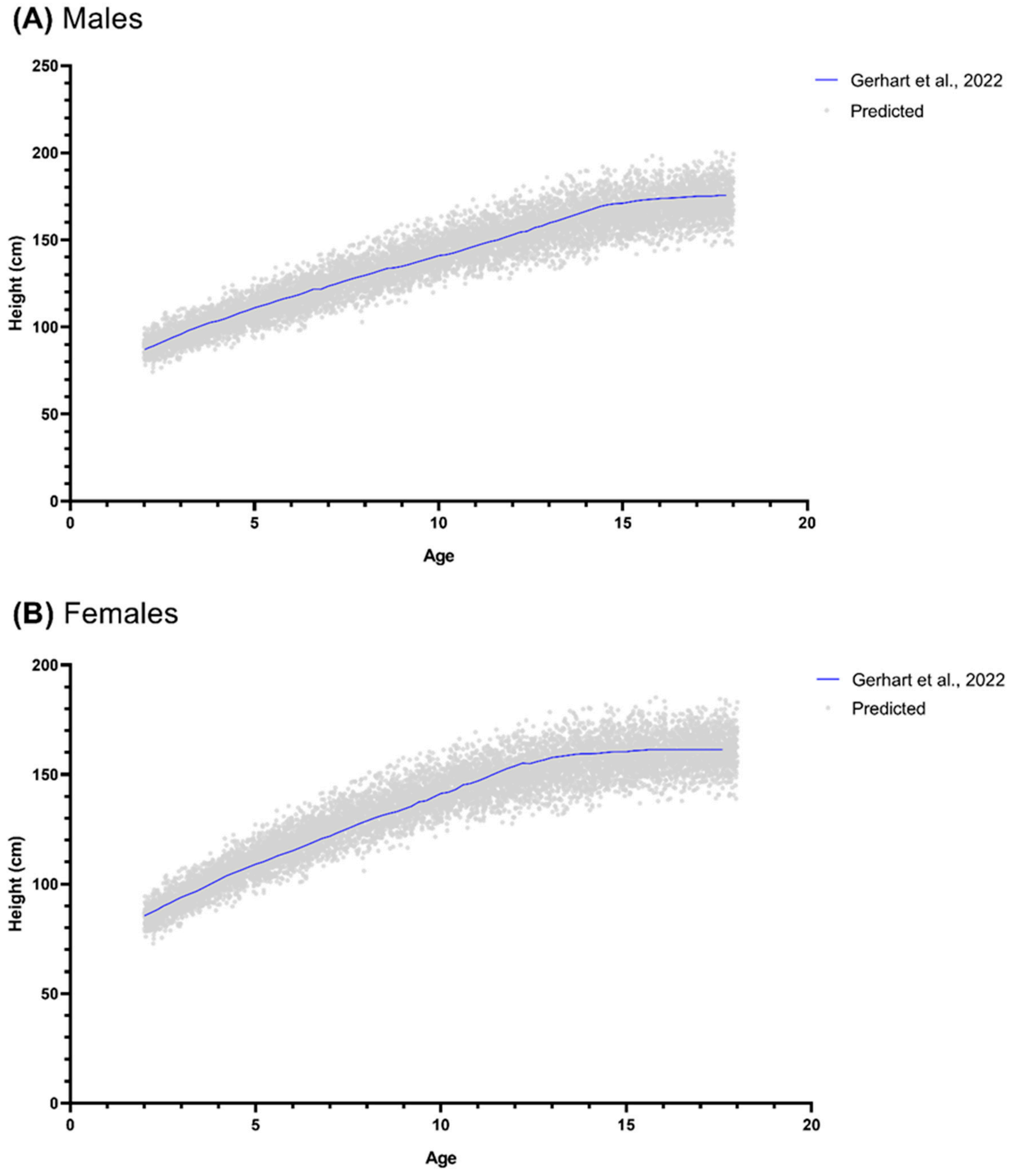

Figure S2. Simulated Height-for-age curve for paediatric obesity from 2 to 18 years old for males (A) and females (B). Gerhart et al. (2022) generated the central tendency of paediatric obesity's height-for-age curve based on the National Health and Nutrition Examination Survey (NHANES) pooled data from 1999 to 2016.

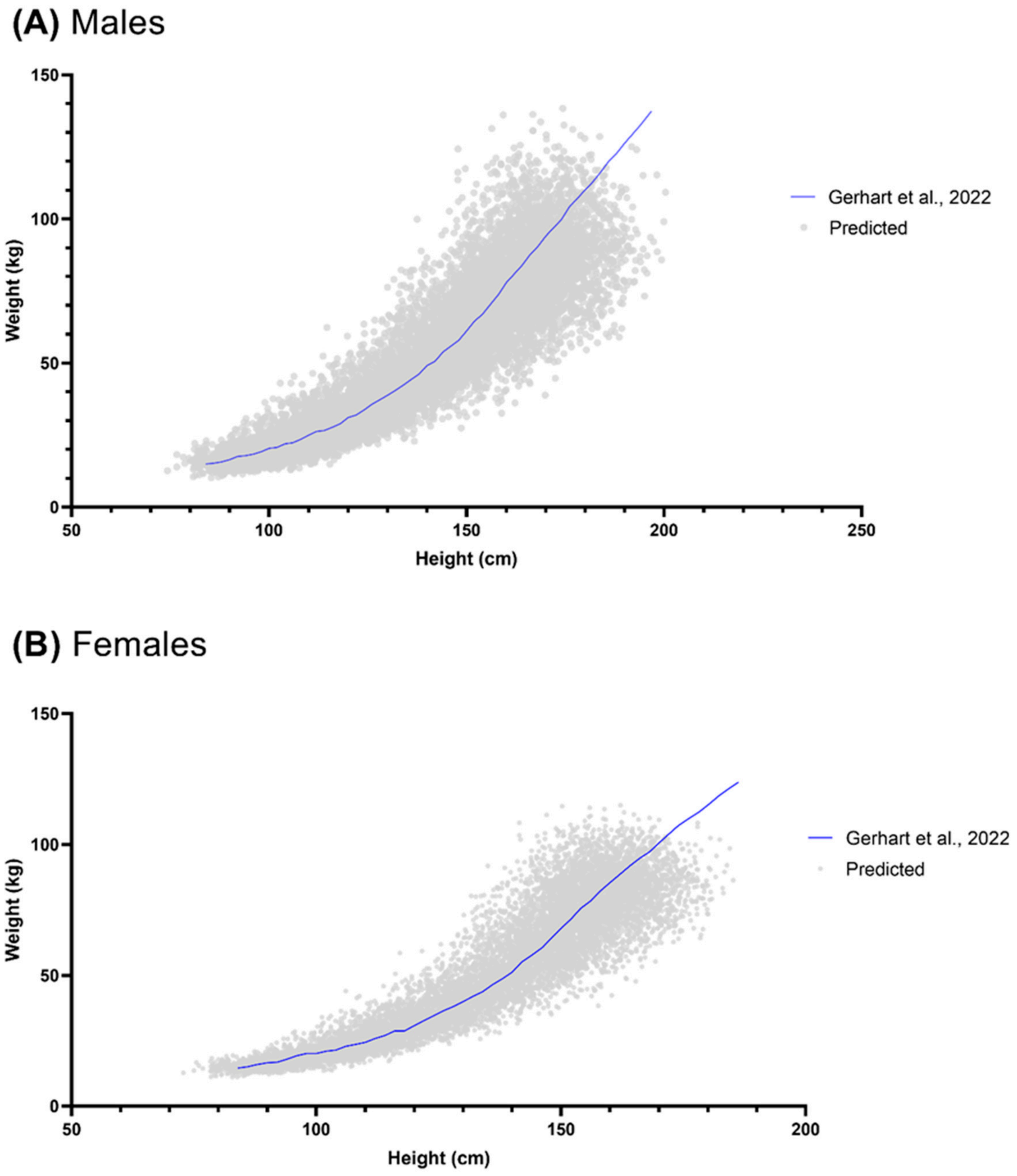

Figure S3. Simulated Weight-for-height curves for paediatric obesity from 2 to 18 years old for males (A) and females (B). Gerhart et al. (2022) generated the central tendency of paediatric obesity's weight-for-age curve based on the NHANES pooled data from 1999 to 2016.

Furthermore, for ages 2 to 5, the reference weight-for-age curves published by WHO and CDC were overlayed on the spread of individual simulated weight-for-height graphs for males and females (Figure S4), reinforcing the validation of simulated weight and height distribution of obese children population (WHO, 2021, CDC, 2017).

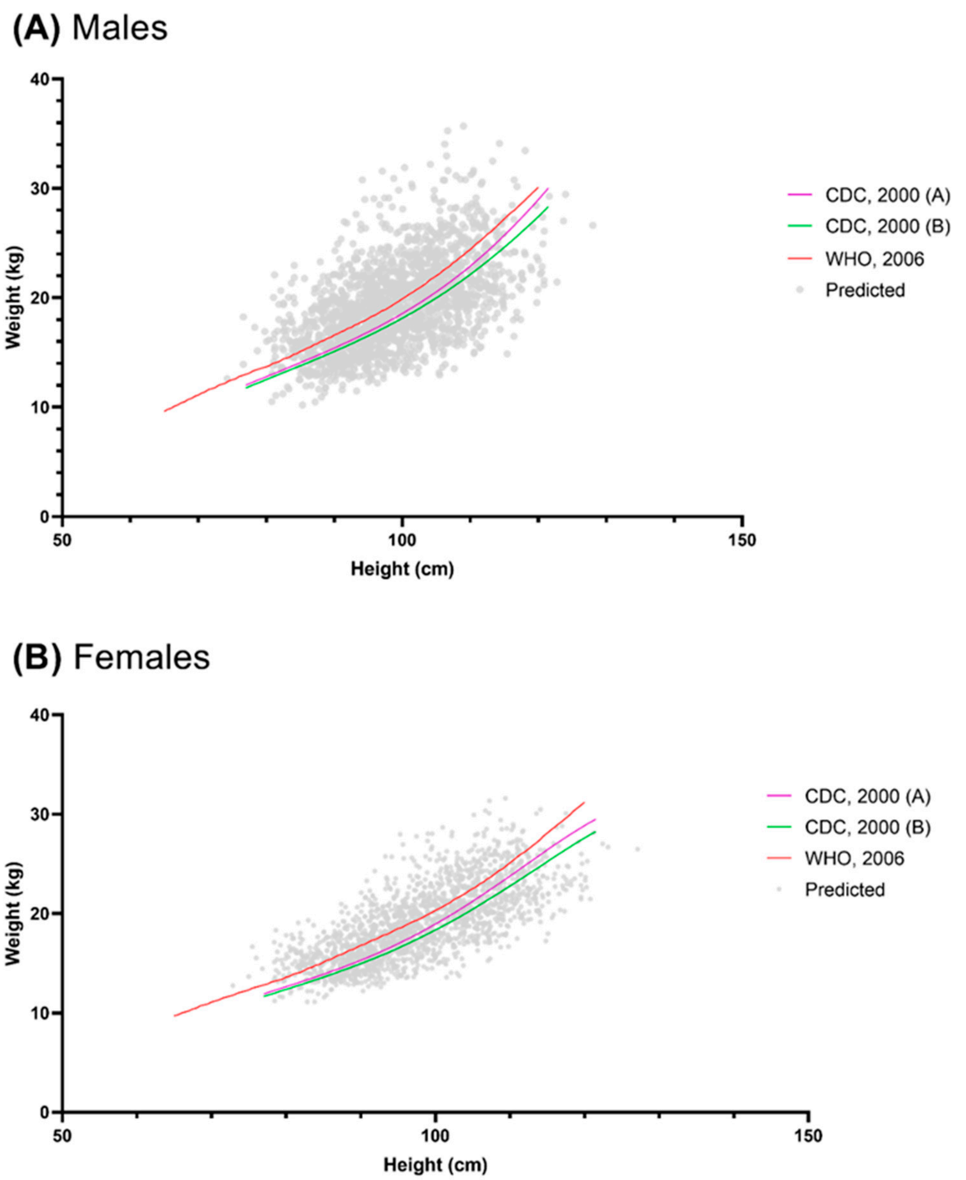

Figure S4. Simulated Weight-for-height curves for paediatric obesity from 2 to 5 years old for males (A) and females (B). The CDC, 2000 (A) Weight-for-height curve is at the 97th percentile (CDC, 2017). The CDC, 2000 (B) Weight-for-height curve is at the 95th percentile, which defines the cut-off curve for obesity in paediatrics (CDC, 2017). The WHO, 2006 Weight-for-age curve is at 3 SD from the median for 2 to 5 years old, which defines the cut-off curve for obesity in paediatrics (WHO, 2021).

Haematocrit-to-age relationship

Haematocrit values correlation with age was predicted using gender-specific mathematical statements as described in Equations (5) and (6);

$$\text{Male } (\%) = 53 - \left(\left(\frac{43 \times \text{age}^{1.12}}{0.05^{1.12} + \text{age}^{1.12}}\right) \times \left(1 + \left(\frac{-0.93 \times \text{age}^{.25}}{0.10^{0.25} + \text{age}^{0.25}}\right)\right)\right) \quad (5)$$

$$\text{Female } (\%) = 53 - \left(\left(\frac{37.4 \times \text{age}^{1.12}}{0.05^{1.12} + \text{age}^{1.12}}\right) \times \left(1 + \left(\frac{-0.80 \times \text{age}^{.25}}{0.10^{0.25} + \text{age}^{0.25}}\right)\right)\right) \quad (6)$$

The distribution of simulated haematocrit values over age fitted within the range of reported values from 8 different references (Figure S5). Besides, the predicted values for both males and females reflected all the published haematocrit values (Table S1), which validated the paediatric obesity population file.

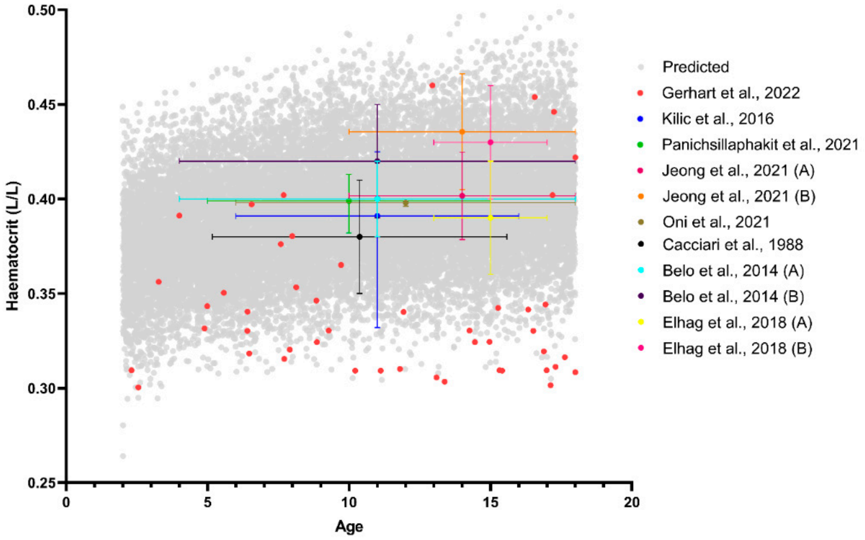

Figure S5. Simulated Haematocrit-to-age relationship for paediatric obesity from 2 to 8 years old (Grey circle). Gerhart et al., 2022 reported individual haematocrit data for obese children from combined clinical trials data represented in the red circles (Gerhart et al., 2022). Jeong et al., 2021 (A) represented data for girls (Jeong et al., 2021). Jeong et al., 2021 (B) represented data for boys (Jeong et al., 2021). Belo et al., 2014 (A) and Belo et al., 2014 (B) represented data for girls and boys, respectively (Belo et al., 2014). Elhag et al., 2018 (A) represented data for girls (Elhag et al., 2018). Elhag et al., 2018 (B) represented data for boys (Elhag et al., 2018). The horizontal lines showed the age range reported for each published study. The coloured circles with the vertical lines are different for each study; Kilic et al., 2016, median with range; Panichsillaphakit et al., 2021, median with interquartile range; Oni et al., 2021, mean with 95% confidence interval; Jeong et al., 2021 mean with standard deviation (SD); Belo et al., 2014, mean with SD; Cacciari et al., 1988, mean with SD; Elhag et al., 2018, mean with SD.

Table S1. Summarised results from literature search for haematocrit values in paediatric obesity

Reference	Age (years)[a]	Number of subjects (n)	Males (%)	Haematocrit (L/L)
Predicted values	2 - 18	10,000	100	0.40 (0.03)[e]
Predicted values	2 - 18	10,000	0	0.38 (0.03)[e]
Kilic et al. (2016)	6 - 16	37	48.64	0.39 (0.33 – 0.43)[b]
Panichsillaphakit et al. (2021)	5 – 15	63	66.78	0.40 (0.38 – 0.41)[c]
Oni et al. (2021)	6 – 19	2,818	53.4	0.40 (0.396 – 0.399)[d]
Jeong et al. (2021)	10 – 18	297	100	0.44 (0.03)[e]
Jeong et al. (2021)	10 – 18	234	0	0.40 (0.02)[e]
Belo et al. (2014)	4 – 18	168	100	0.42 (0.03)[e]
Belo et al. (2014)	4 – 18	182	0	0.40 (0.02)[e]
Cacciari et al. (1988)	5.17 – 15.58	43	65	0.38 (0.03)[e]
Elhag et al. (2018)	13 – 17	36	100	0.43 (0.03)[e]
Elhag et al. (2018)	13 - 17	43	0	0.39 (0.03)[e]

[a] range; [b] median (min-max); [c] median (interquartile range); [d] mean (95% confidence interval); [e] mean (SD)

Serum albumin-to-age relationship

As no significant difference was reported in human serum albumin values between genders (Gerhart et al., 2022), the relationship for the range of age between 2 to 18 years old was predicted based on the general mathematical equation (7);

$$\text{Serum albumin (g/L)} = 33.746 + (1.1287 \times \ln(365 \times age)) \quad (7)$$

The spread of simulated serum albumin over age values was within the wide range of published values from 7 references (Figure S6). Additionally, the predicted values (27.62 – 58.19 g/L) are echoed with the published reference values (Table S2) and simulated values by Gerhart et al. (2022). Therefore, it validated the simulated serum albumin values for the paediatric obesity population developed in this study.

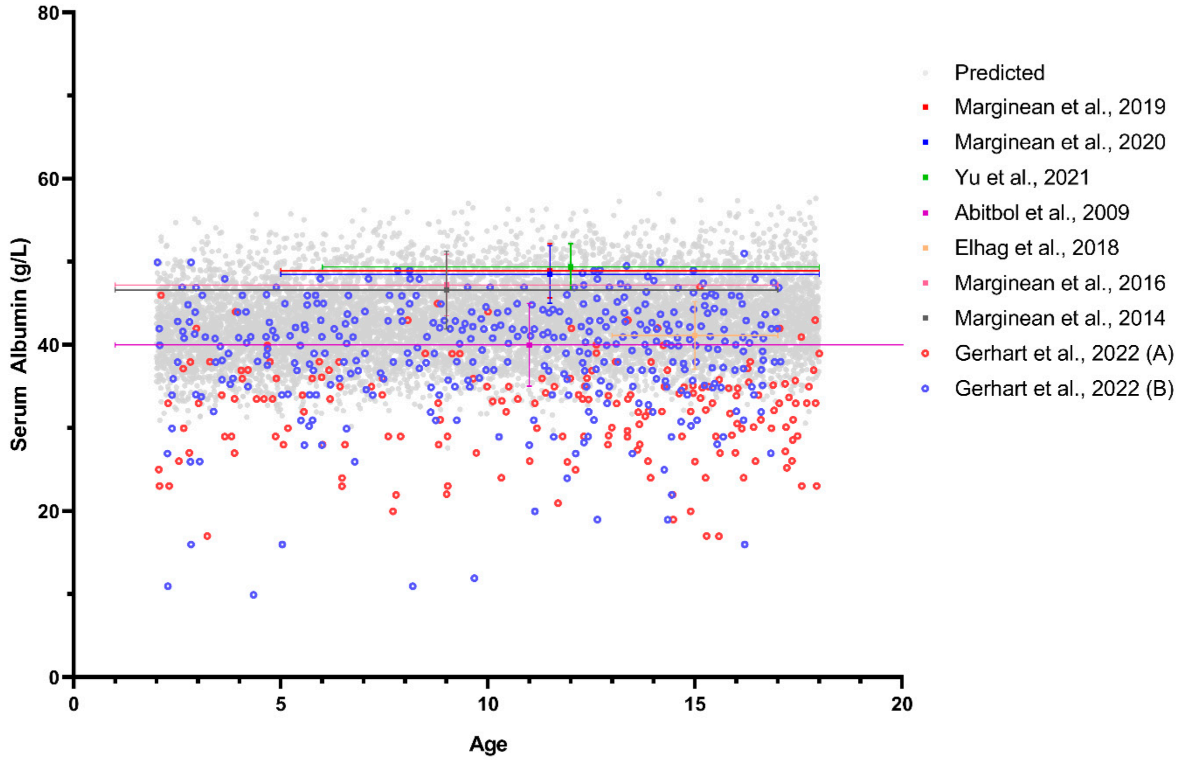

Figure S6. Predicted Serum albumin-to-age relationship for paediatric obesity from 2 to 18 years old (Grey circle). Gerhart et al., 2022 (A) reported individual serum albumin data for children with obesity from combined clinical trials data in red circles (Gerhart et al., 2022). Gerhart et al., 2022 (B) reported individual data for paediatric obesity from the Paediatric Trial Network (PTN) data repository (Gerhart et al., 2022). The horizontal lines showed the age range reported for each published study. The coloured squares with vertical lines represented the mean with SD.

Table S2. Summarised results from literature search for serum albumin values in paediatric obesity

Reference	Age (years)[a]	Number of subjects (n)	Males (%)	Serum Albumin (g/L)
Predicted values	2 - 18	20,000	50	42.80 (4.27)[b]
Yu et al. (2021)	6 – 18	449	62.81	49.40 (2.80)[b]
Elhag et al. (2018)	13 – 17	79	45	41.14 (4.06)[b]
Abitbol et al. (2009)	1 – 21	22	50	40.00 (5.0)[b]
Marginean et al. (2014)	1 – 18	102	57.84	46.60 (4.70)[b]
Marginean et al. (2016)	1 – 18	121	53.72	47.20 (3.70)[b]
Marginean et al. (2019)	5 – 18	77	NR	48.92 (3.26)[b]
Marginean et al. (2020)	5 – 18	91	NR	48.48 (3.46)[b]

NR, not reported; [a] range; [b] mean (SD)

Alpha-1-acid glycoprotein (AGP)-to-age relationship

A similar pattern can be seen in AGP-to-age correlation as another protein-binding component in blood. One polynomial mathematical equation (8) to describe the correlation with age from 2 to 18 years old;

$$\text{AGP}\ \left(\frac{g}{L}\right) = \frac{0.887 \times (365 \times age)^{0.38}}{(8.89^{0.38} + (365 \times age)^{0.38}} \quad (8)$$

The distribution of predicted AGP values was within the broad range of AGP values observed from 4 different studies (Figure S7). Furthermore, the predicted values were comparable with the observed values, thus validating the obese children population file (Table S3).

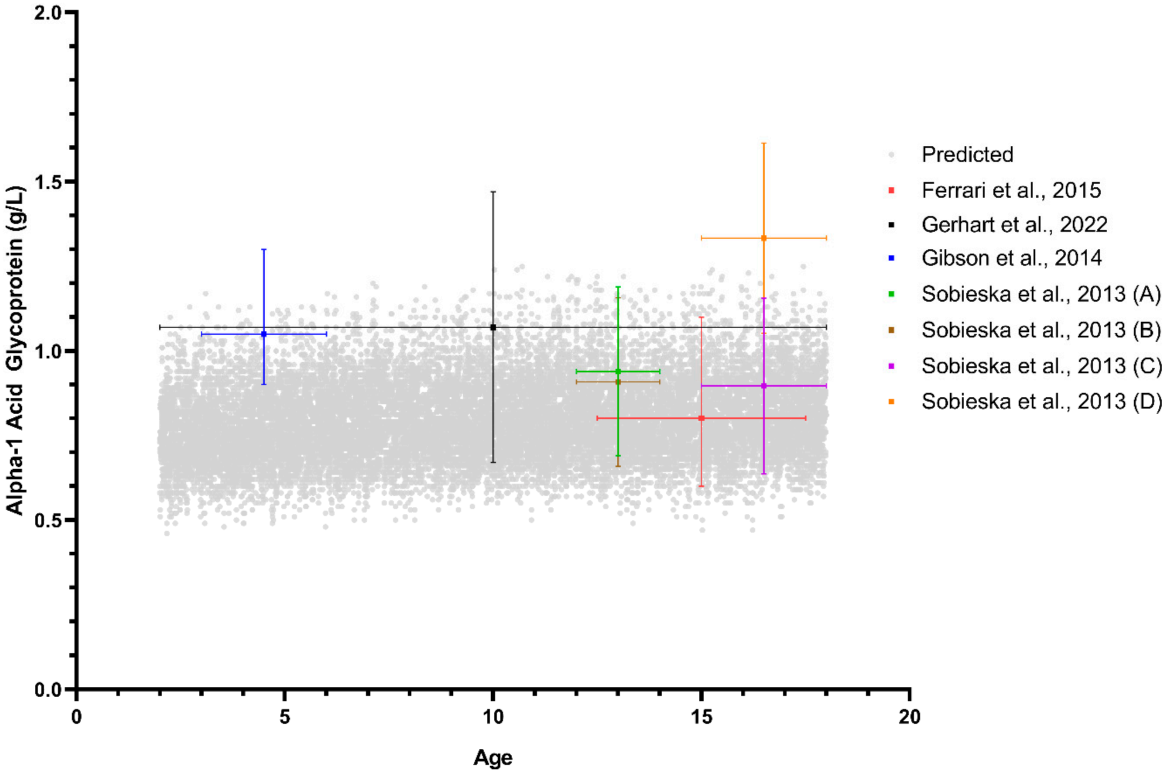

Figure S7. Predicted AGP-to-age relationship for paediatric obesity from 2 to 18 years old (Grey circle). Sobieska et al., 2013 (A) represented data for boys aged 12 to 14 (Sobieska et al., 2013). Sobieska et al., 2013 (B) represented data for girls aged 12 to 14 (Sobieska et al., 2013). Sobieska et al., 2013 (C) represented data for boys 15 to 18 years old (Sobieska et al., 2013). Sobieska et al., 2013 (D) represented data for girls 15 to 18 years old (Sobieska et al., 2013). The horizontal lines showed the age range reported for each published study. The coloured squares with vertical lines represented the mean with SD for Gerhart et al. (2022) and Sobieska et al. (2013). The coloured square with vertical lines represented the median with range for Gibson et al. (2014) and Ferrari et al. (2015).

Table S3. Summarised results from literature search for AGP values in paediatric obesity

Reference	Age (years)[a]	Number of subjects (n)	Males (%)	AGP (g/L)
Predicted values	2 - 18	20,000	50	0.80 (0.10)[c]
Gerhart et al. (2022)[b]	2 – 18	32,001	50	1.07 (0.40)[c]
Sobieska et al. (2013)	12 – 14	28	100	0.94 (0.25)[c]
Sobieska et al. (2013)	12 – 14	23	0	0.91 (0.25)[c]
Sobieska et al. (2013)	15 – 18	33	100	0.90 (0.26)[c]
Sobieska et al. (2013)	15 – 18	40	0	1.33 (0.28)[c]
Gibson et al. (2014)	3 – 6	49	NR	1.05 (0.90 – 1.30)[d]
Ferrari et al. (2015)	12.5 – 17.5	876	46	0.80 (0.60 – 1.10)[d]

NR, not reported; [a] range; [b] Gerhart et al., 2022 AGP values are the simulated values for paediatric obesity; [c] mean (SD); [d] median (range)

Glomerular filtration rate (GFR)-to-age relationship

Since GFR is the function of body surface area (BSA) as described in mathematical equation (9), the GFR increases as age increases;

$$GFR\left(\frac{mL}{min}\right) = -17.74 + 99.054(BSA) - 6.1604(BSA)^2 \tag{9}$$

The absolute GFR was comparable with the reported values by Correia-Costa et al. (2016) for paediatric obesity ages 8 to 9 (Figure **Error! Reference source not found.**S8 and Table S4). Additionally, BSA-adjusted GFR for obese children aged 4 – 18 years old was compared with values published by Duzova et al. (2013) and Goknar et al. (2015). The predicted values in virtual obese children reflected the published values (Table S4) despite a broader range at 16 to 18 years old in the simulated values (Figure S8). Considering both predicted absolute and BSA-adjusted GFR were in line with observed values, it validated the paediatric obesity population file.

Table S4. Summarised results from literature search for GFR values in paediatric obesity

Reference	Age (years)[a]	Number of subjects (n)	Males (%)	GFR (mL/min)
Predicted values	8 – 9	1,230	50	103.50 (18.19)[c]
Correia-Costa et al. (2016)	8 – 9	61	66	120.80 (21.50)[c]

Predicted values	5 – 18	16,274	50	137.40 (24.72)[d]
Duzova et al. (2013)	5 – 18	318	NR	122.70 (21.60)[d]
Predicted values	4 – 16	15,104	50	132.70 (20.44)[d]
Goknar et al. (2015)	4 – 16	84	54.76	152.22 (23.94)[d]

NR, not reported; [a] range; [b] Gerhart et al., 2022 GFR values are the predicted values for paediatric obesity; [c] mean (SD); a unit of mL/min/1.73m^2, presented as mean (SD).

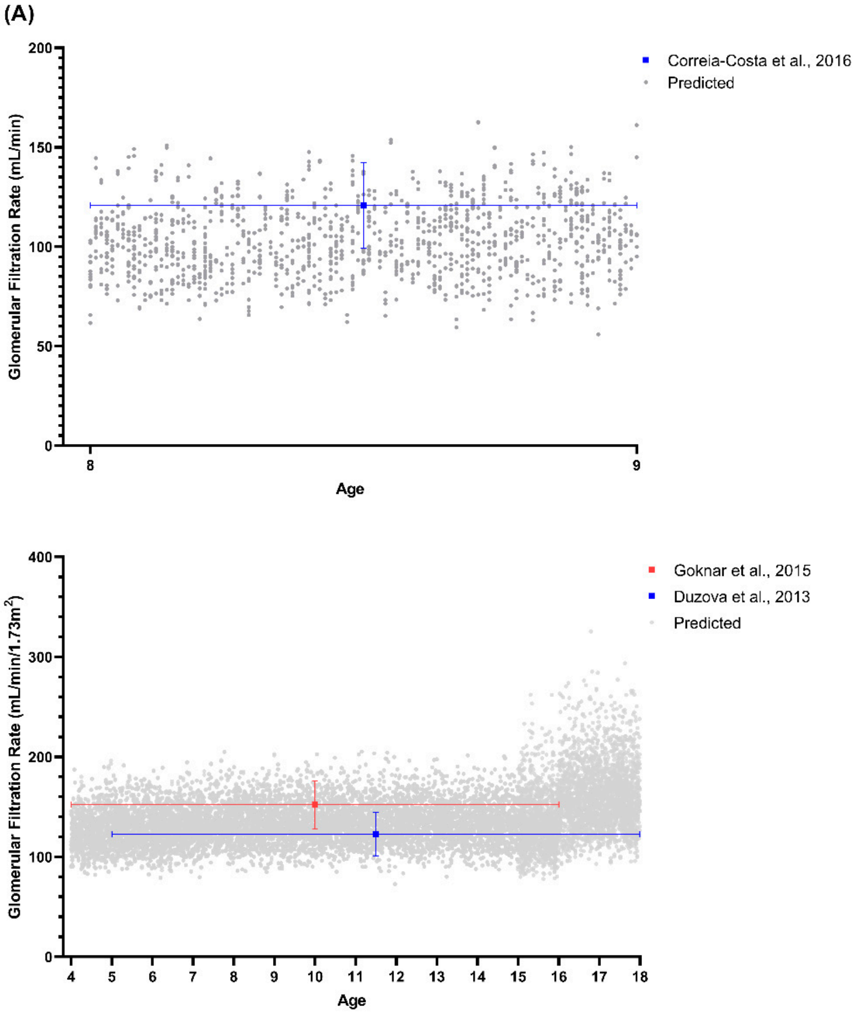

Figure S8. Absolute GFR(mL/min)-to-age relationship for paediatric obesity from 8 to 9 years old (A) and BSA-adjusted GFR(mL/min/1.73m^2)-to-age correlation for paediatric obesity from 8 to 9 years old (B). Grey circles are the predicted value. The horizontal lines showed the age range reported for each published study. The coloured squares with vertical lines represented the mean with SD.

2. Supplementary figures

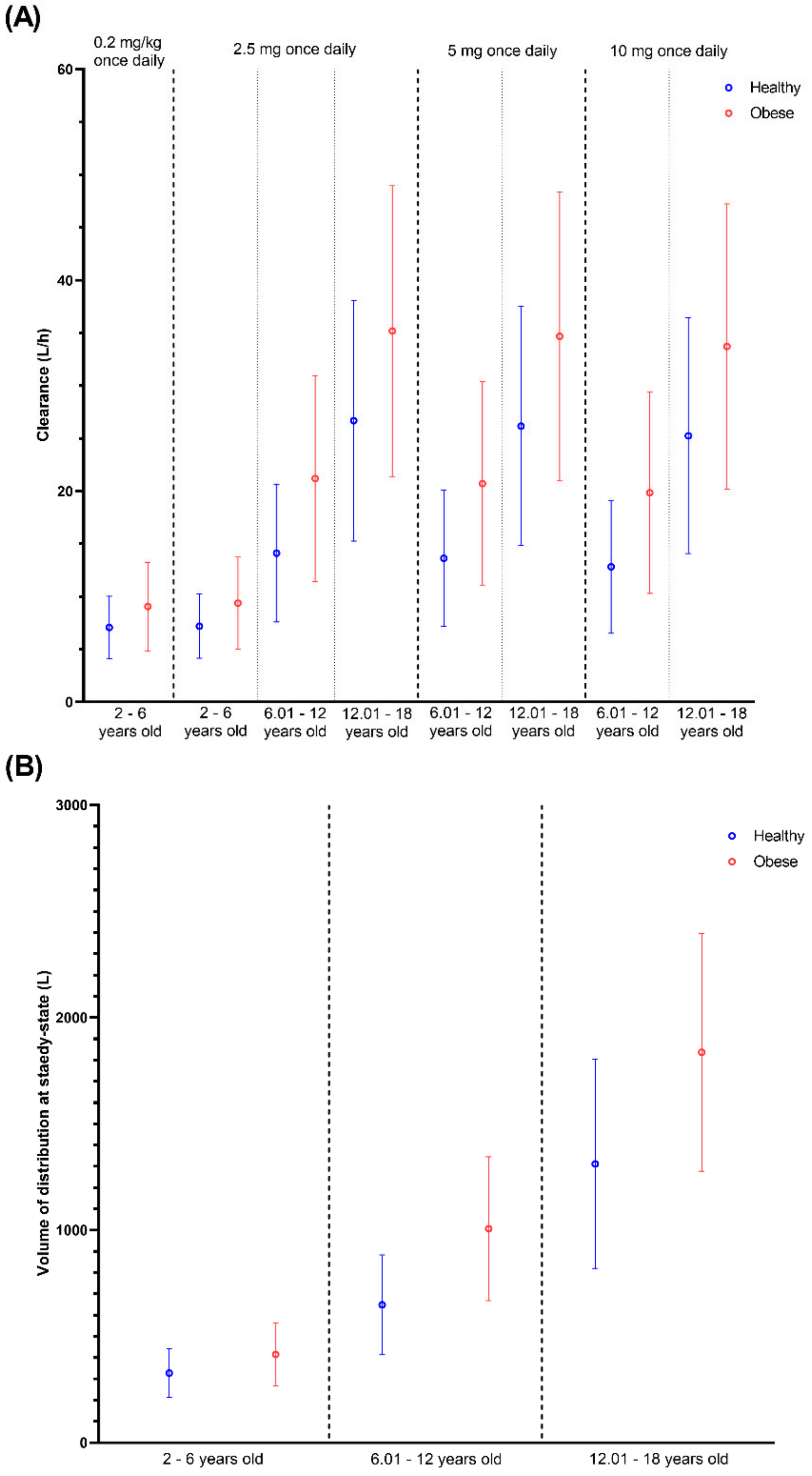

Figure S9. Comparison of predicted clearance (A) and volume of distribution (B) at steady-state for healthy and obese paediatric doses. The coloured circles represent the mean, and the horizontal lines represent the standard deviations.

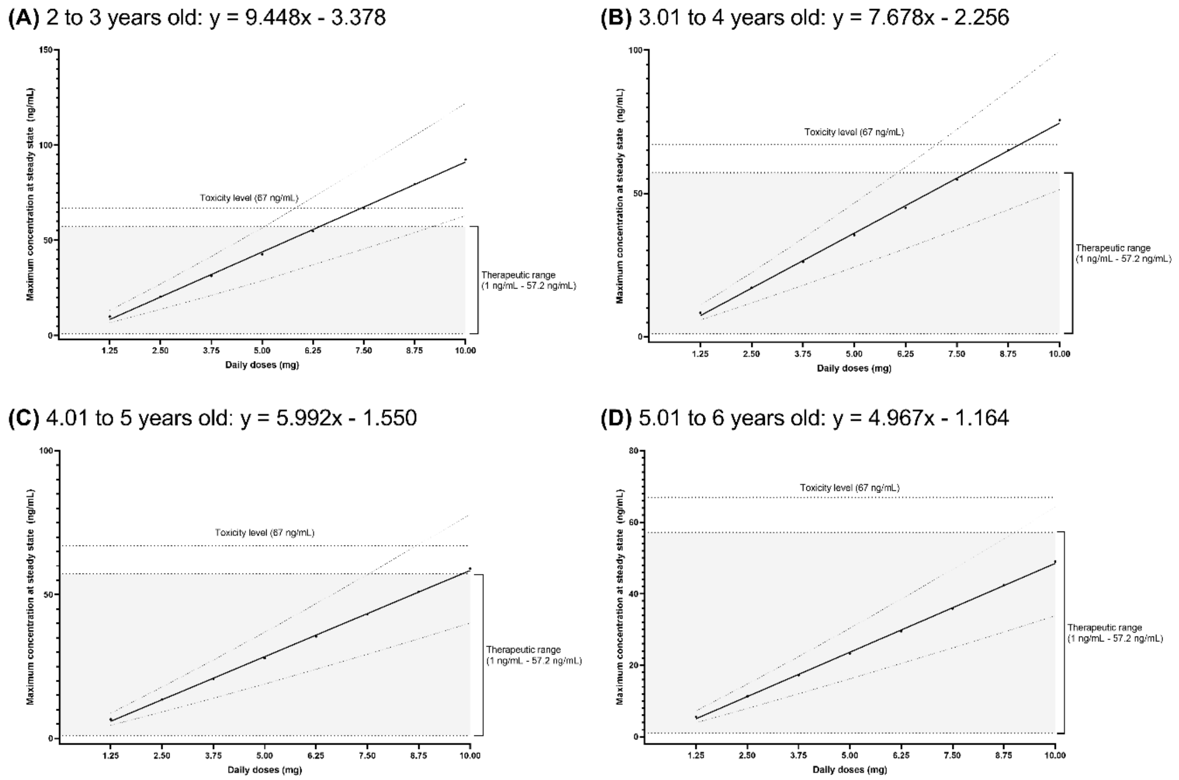

Figure S10. Predicted maximum concentrations (C_{max}) versus daily doses for age group 2 to 6 years old. Solid lines represent the means, dotted lines represent the SD, and grey area is the therapeutic range for amlodipine (1 ng/mL – 57.2 ng/mL).

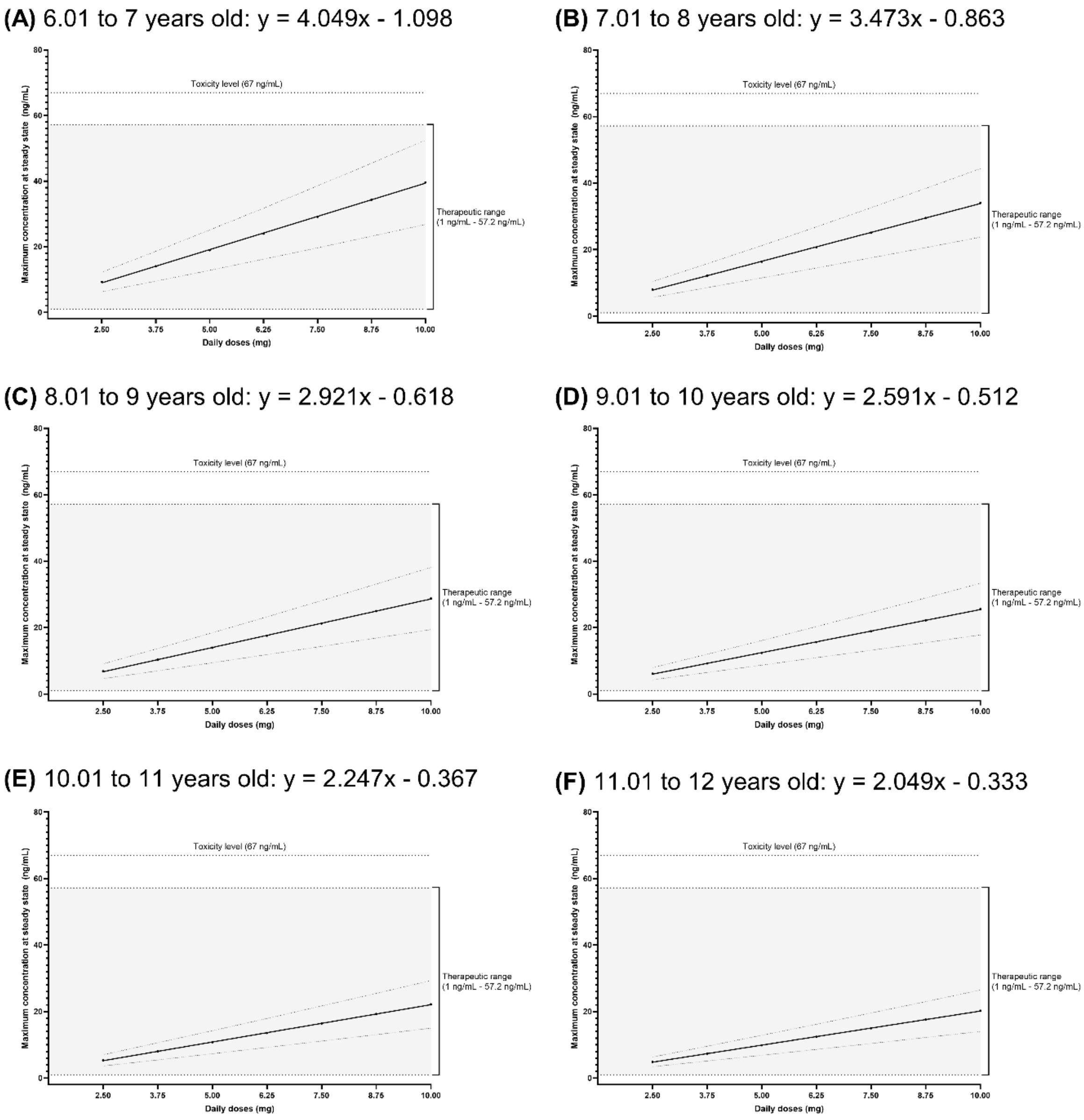

Figure S11. Predicted C_{max} versus daily doses for age group 6.01 to 12 years old. Solid lines represent the means, dotted lines represent the SD, and grey area is the therapeutic range for amlodipine (1 ng/mL – 57.2 ng/mL).

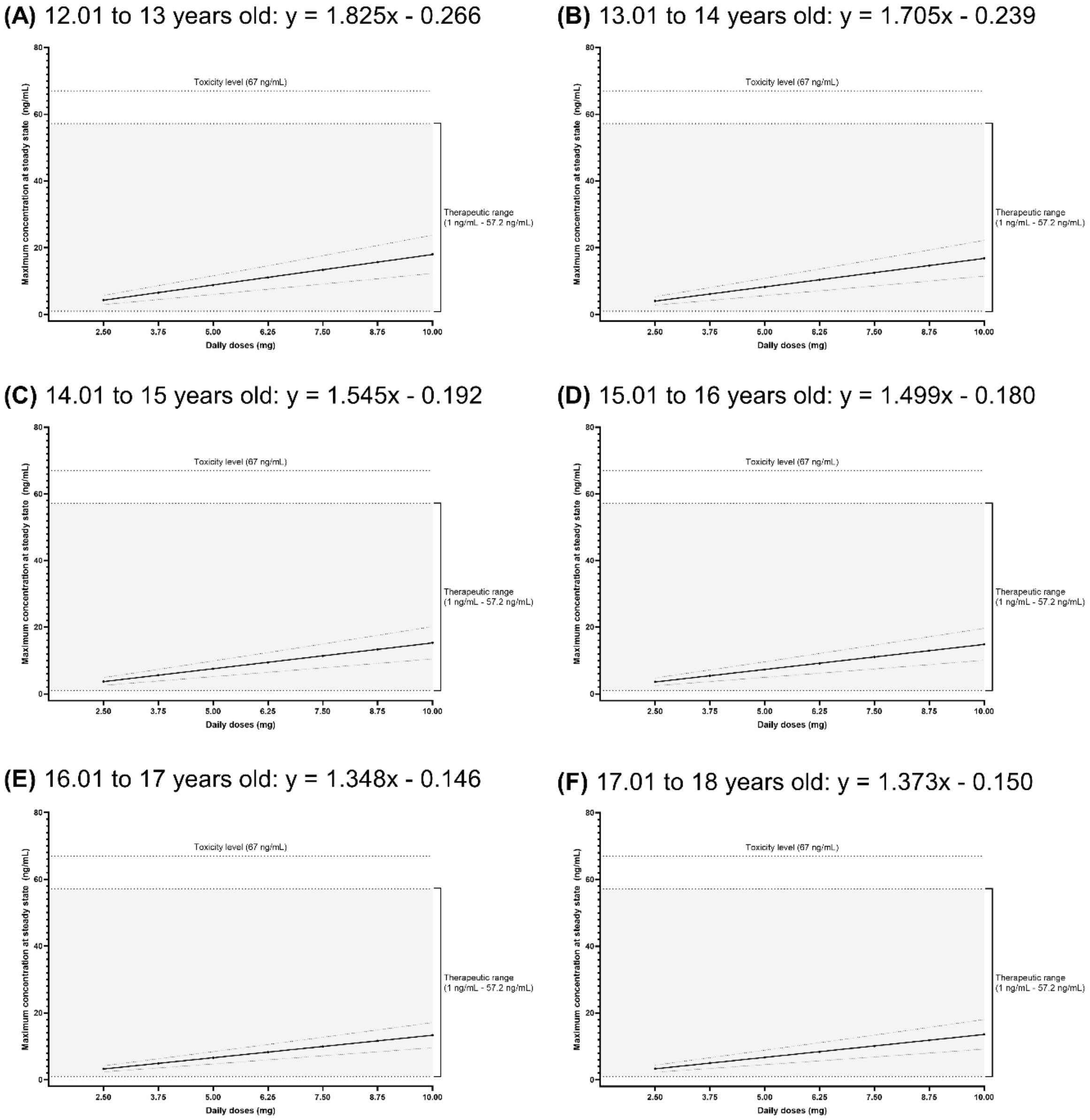

Figure S12. Predicted C_{max} versus daily doses for age group 12.01 to 18 years old. Solid lines represent the means, dotted lines represent the SD, and grey area is the therapeutic range for amlodipine (1 ng/mL – 57.2 ng/mL).

Dose		2	3	4	5	6	7	8	9	10	11	12	13	14	15	16	17	18
0.10 mg/kg	C_{max} (ng/mL)	12.48 ± 3.34	12.22 ± 3.26	11.82 ± 3.42	11.4 ± 3.01	11.14 ± 3.23	10.88 ± 2.94	10.76 ± 3.14	10.63 ± 2.95	10.59 ± 3.12	10.56 ± 3.01							
	C_{min} (ng/mL)	8.55 ± 3.1	7.84 ± 2.52	8.21 ± 3.12	7.46 ± 2.34	7.82 ± 2.96	7.21 ± 2.29	7.61 ± 2.88	7.11 ± 2.3	7.53 ± 2.85	7.12 ± 2.34							
	$AUC_{0\text{-}24ss}$ (ng/mL.h)	252.8 ± 78.77	240.16 ± 67.56	241.65 ± 79.81	226.58 ± 63.06	229.19 ± 75.7	217.39 ± 62.04	222.11 ± 73.69	213.28 ± 62.38	219.12 ± 73.13	212.5 ± 63.85							
	% dose > 10 mg/day	0	0	0	0	0	0	0	0	0	0							
	% C_{max} > 67 ng/mL	0	0	0	0	0	0	0	0	0	0							
	1 ng/mL < % C_{max} < 57.2 ng/mL	100	100	100	100	100	100	100	100	100	100							
0.40 mg/kg	C_{max} (ng/mL)	54.13 ± 14.41	53.06 ± 14.32	51.19 ± 14.7	49.34 ± 13.27	48.09 ± 13.83	46.98 ± 12.97	46.41 ± 13.46	45.9 ± 13	45.68 ± 13.37	45.6 ± 13.3							
	C_{min} (ng/mL)	38.14 ± 13.38	35.23 ± 11.38	36.48 ± 13.44	33.32 ± 10.56	34.6 ± 12.72	32.06 ± 10.33	33.59 ± 12.38	31.58 ± 10.35	33.21 ± 12.26	31.6 ± 10.56							
	$AUC_{0\text{-}24ss}$ (ng/mL.h)	1113.44 ± 339.84	1060.95 ± 302.82	1060.97 ± 344.01	996.25 ± 283.2	1002.63 ± 324.92	953.42 ± 278.05	970.28 ± 316.62	934.52 ± 279.54	956.79 ± 314.49	931.11 ± 286.28							
	% dose > 10 mg/day	0	0	0	7.55	8.51	28.3	65.96	71.7	93.62	96.27							
	% C_{max} > 67 ng/mL	25	21.15	14.89	13.21	10.64	11.32	10.64	9.43	8.51	11.32							
	1 ng/mL < % C_{max} < 57.2 ng/mL	62.5	69.23	68.09	77.36	74.47	77.36	80.85	77.36	80.85	77.36							
2.50 mg OD	C_{max} (ng/mL)	23.63 ± 7.7	21.2 ± 6.34	17.72 ± 5.82	15.59 ± 4.53	13.25 ± 4.42	11.89 ± 3.5	10.37 ± 3.5	9.43 ± 2.79	8.26 ± 2.91	7.44 ± 2.24	6.47 ± 2.35	5.85 ± 1.8	5.15 ± 1.85	4.84 ± 1.67	4.32 ± 1.26	4.46 ± 1.61	
	C_{min} (ng/mL)	16.36 ± 6.82	13.74 ± 4.93	12.38 ± 5.18	10.26 ± 3.56	9.34 ± 3.95	7.91 ± 2.76	7.35 ± 3.13	6.32 ± 2.23	5.88 ± 2.57	5.02 ± 1.8	4.62 ± 2.07	3.96 ± 1.43	3.68 ± 1.62	3.25 ± 1.3	3.02 ± 1.06	3.01 ± 1.23	
	$AUC_{0\text{-}24ss}$ (ng/mL.h)	481.54 ± 177.36	418.78 ± 132.96	363.52 ± 134.44	310.64 ± 96.21	273.3 ± 102.44	238.03 ± 74.71	214.28 ± 81.21	189.28 ± 60.14	170.97 ± 67.09	149.71 ± 48.45	134.08 ± 54.14	117.79 ± 38.82	106.75 ± 42.6	97 ± 35.35	88.6 ± 28.07	89.58 ± 33.71	
	% C_{max} > 67 ng/mL	0	0	0	0	0	0	0	0	0	0	0	0	0	0	0	0	
	1 ng/mL < % C_{max} < 57.2 ng/mL	100	100	100	100	100	100	100	100	100	100	100	100	100	100	100	98.11	
5.00 mg OD	C_{max} (ng/mL)	49.66 ± 16.21	44.44 ± 13.39	36.9 ± 12.12	32.36 ± 9.5	27.37 ± 9.13	24.51 ± 7.27	21.28 ± 7.21	19.33 ± 5.78	16.87 ± 5.95	15.19 ± 4.6	13.16 ± 4.8	11.88 ± 3.68	10.45 ± 3.76	9.8 ± 3.41	8.74 ± 2.56	9.04 ± 3.27	
	C_{min} (ng/mL)	34.97 ± 14.39	29.38 ± 10.59	26.13 ± 10.81	21.63 ± 7.55	19.49 ± 8.18	16.48 ± 5.8	15.21 ± 6.44	13.08 ± 4.64	12.1 ± 5.28	10.32 ± 3.72	9.45 ± 4.22	8.1 ± 2.95	7.5 ± 3.3	6.62 ± 2.65	6.14 ± 2.14	6.11 ± 2.51	
	$AUC_{0\text{-}24ss}$ (ng/mL.h)	1021.29 ± 374.21	886.59 ± 284.11	762.29 ± 280.81	649.87 ± 203.37	567.53 ± 212.18	493.62 ± 156.35	441.92 ± 167.35	390.04 ± 125.01	350.68 ± 137.65	306.81 ± 100.07	273.65 ± 110.6	240.2 ± 79.64	217.04 ± 86.7	197.06 ± 72.21	179.66 ± 56.95	181.8 ± 68.76	
	% C_{max} > 67 ng/mL	5.77	3.85	2.13	0	0	0	0	0	0	0	0	0	0	0	0	0	
	1 ng/mL < % C_{max} < 57.2 ng/mL	88.46	82.69	93.62	95.75	100	100	100	100	100	100	100	100	100	100	100	100	
		2	3	4	5	6	7	8	9	10	11	12	13	14	15	16	17	18

Age

Figure S13. Summary of pharmacokinetic parameters at steady-state in healthy paediatric from 2 to 18 years old. C_{max}, maximum concentration; C_{min}, minimum concentration; $AUC_{0\text{-}24ss}$, area-under-the-curve at steady-state; 67 ng/mL, toxic level; 1 ng/mL to 57.2 ng/mL, therapeutic concentration.

Dose		Age										
		2	3	4	5	6	7	8	9	10	11	12
0.10 mg/kg	C_{max} (ng/mL)	12.53 ± 3.44	12.25 ± 3.27	12.02 ± 3.22	11.93 ± 3.19	11.83 ± 3.28	11.84 ± 3.21	11.81 ± 3.35	11.86 ± 3.29	11.81 ± 3.4	11.86 ± 3.35	
	C_{min} (ng/mL)	8.57 ± 3.18	18.65 ± 5	8.34 ± 3.01	7.83 ± 2.45	8.31 ± 3.03	7.88 ± 2.52	8.36 ± 3.08	7.97 ± 2.58	8.4 ± 3.11	8.03 ± 2.63	
	$AUC_{0\text{-}24ss}$ (ng/mL.h)	253.57 ± 80.9	25.22 ± 6.78	245.38 ± 76.38	236.85 ± 66.43	243.19 ± 77.23	236.73 ± 67.93	243.87 ± 78.98	238.22 ± 69.85	244.49 ± 80	238.99 ± 71.31	
	% dose > 10 mg/day	0	0	0	0	0	0	0	0	0	0	
	% C_{max} > 67 ng/mL	0	0	0	0	0	0	0	0	0	0	
	1 ng/mL < % C_{max} < 57.2 ng/mL	100	100	100	100	100	100	100	100	100	100	
0.20 mg/kg	C_{max} (ng/mL)	25.8 ± 7.07	25.22 ± 6.78	24.74 ± 6.61	24.55 ± 6.62	24.33 ± 6.73	24.36 ± 6.66	24.3 ± 6.89	24.42 ± 6.84	24.3 ± 6.99	24.42 ± 6.97	
	C_{min} (ng/mL)	17.82 ± 6.54	16.36 ± 5.19	17.32 ± 6.18	16.29 ± 5.12	17.25 ± 6.23	16.4 ± 5.27	17.36 ± 6.35	16.59 ± 5.41	17.44 ± 6.41	16.71 ± 5.52	
	$AUC_{0\text{-}24ss}$ (ng/mL.h)	524.99 ± 166.68	497.85 ± 140.33	507.63 ± 156.93	490.39 ± 138.82	502.89 ± 158.82	490.12 ± 142.05	504.31 ± 162.57	493.32 ± 146.13	505.62 ± 164.78	495.01 ± 149.29	
	% dose > 10 mg/day	0	0	0	0	0	0	12.77	47.17	74.47	88.68	
	% C_{max} > 67 ng/mL	0	0	0	0	0	0	0	0	0	0	
	1 ng/mL < % C_{max} < 57.2 ng/mL	100	100	100	100	100	100	100	100	100	100	
0.30 mg/kg	C_{max} (ng/mL)	39.75 ± 10.86	38.88 ± 10.49	38.1 ± 10.13	37.83 ± 10.26	37.47 ± 10.31	37.55 ± 10.34	37.43 ± 10.57	37.64 ± 10.62	37.43 ± 10.73	37.65 ± 10.83	
	C_{min} (ng/mL)	27.71 ± 10.04	45.94 ± 12.42	26.91 ± 9.48	25.37 ± 8.01	26.78 ± 9.56	25.53 ± 8.24	26.94 ± 9.75	25.82 ± 8.46	27.07 ± 9.85	26 ± 8.63	
	$AUC_{0\text{-}24ss}$ (ng/mL.h)	812.81 ± 256	53.16 ± 14.38	785.52 ± 240.61	759.68 ± 216.51	777.91 ± 243.67	759.24 ± 221.65	780.12 ± 249.6	764.36 ± 228.11	782.22 ± 253.11	767.14 ± 233.2	
	% dose > 10 mg/day	0	0	0	9.43	44.68	67.93	95.75	100	100	100	
	% C_{max} > 67 ng/mL	0	0	0	0	0	0	0	0	0	0	
	1 ng/mL < % C_{max} < 57.2 ng/mL	93.75	96.15	95.75	94.34	91.49	94.34	91.49	94.34	91.49	92.45	
0.40 mg/kg	C_{max} (ng/mL)	54.32 ± 14.74	53.16 ± 14.38	52.06 ± 13.74	51.71 ± 14.08	51.2 ± 13.99	51.33 ± 14.21	51.13 ± 14.35	51.47 ± 14.6	51.15 ± 14.57	51.51 ± 14.9	
	C_{min} (ng/mL)	38.19 ± 13.64	35.24 ± 11.24	37.05 ± 12.87	35 ± 11.08	36.85 ± 12.97	35.22 ± 11.4	37.07 ± 13.24	35.62 ± 11.7	37.24 ± 13.39	35.86 ± 11.94	
	$AUC_{0\text{-}24ss}$ (ng/mL.h)	1115.57 ± 347.7	1060.93 ± 301.79	1077.74 ± 326.48	1043.58 ± 298.7	1067.01 ± 330.73	1042.99 ± 305.89	1070.04 ± 338.98	1050.24 ± 314.9	1073.03 ± 343.88	1054.28 ± 322.11	
	% dose > 10 mg/day	0	1.92	23.4	62.26	95.75	100	100	100	100	100	
	% C_{max} > 67 ng/mL	18.75	15.38	12.77	15.09	14.89	15.09	14.89	16.98	17.02	16.98	
	1 ng/mL < % C_{max} < 57.2 ng/mL	62.5	63.46	61.7	69.81	68.09	71.7	68.09	69.81	68.09	69.81	
		2	3	4	5	6	7	8	9	10	11	12
		Age										

Figure S14. Summary of pharmacokinetic parameters at steady-state in paediatric obesity from 2 to 18 years old administered with weight-based dose. C_{max}, maximum concentration; C_{min}, minimum concentration; $AUC_{0\text{-}24ss}$, area-under-the-curve at steady-state; 67 ng/mL, toxic level; 1 ng/mL to 57.2 ng/mL, therapeutic concentration.

		Age															
Dose		2	3	4	5	6	7	8	9	10	11	12	13	14	15	16	17 18
2.50 mg OD	C_{max} (ng/mL)	20.41 ± 6.65	17.06 ± 5.31	13.52 ± 4.35	11.32 ± 3.4	9.28 ± 3	8.02 ± 2.37	6.83 ± 2.21	6.09 ± 1.82	5.32 ± 1.71	4.87 ± 1.47	4.36 ± 1.38	4.08 ± 1.28	3.72 ± 1.17	3.61 ± 1.16	3.26 ± 0.91	3.32 ± 1.07
	C_{min} (ng/mL)	14.06 ± 5.84	11.02 ± 4.02	9.39 ± 3.85	7.46 ± 2.63	6.51 ± 2.67	5.35 ± 1.89	4.82 ± 1.97	4.09 ± 1.45	3.77 ± 1.53	3.29 ± 1.17	3.1 ± 1.23	2.77 ± 1	2.64 ± 1.04	2.45 ± 0.91	2.29 ± 0.8	2.26 ± 0.83
	$AUC_{0\text{-}24ss}$ (ng/mL.h)	414.67 ± 152.39	335.94 ± 110.08	276.27 ± 100.23	225.22 ± 71.65	190.75 ± 69.33	160.49 ± 50.86	140.8 ± 51.29	122.18 ± 39.05	109.93 ± 39.72	97.98 ± 31.69	90.19 ± 32.09	82.24 ± 27.24	76.91 ± 27.13	72.79 ± 24.58	67.11 ± 20.77	67 ± 22.66
	% C_{max} > 67 ng/mL	0	0	0	0	0	0	0	0	0	0	0	0	0	0	0	0
	1 ng/mL < % C_{max} < 57.2 ng/mL	100	97.92	100	100	100	100	100	100	100	100	100	100	100	100	100	100
3.75 mg OD	C_{max} (ng/mL)	31.32 ± 10.22	26.11 ± 8.18	20.61 ± 6.64	17.23 ± 5.2	14.08 ± 4.56	12.16 ± 3.61	10.33 ± 3.35	9.2 ± 2.76	8.04 ± 2.59	7.35 ± 2.23	6.58 ± 2.08	6.16 ± 1.93	5.6 ± 1.76	5.44 ± 1.75	4.91 ± 1.38	5 ± 1.62
	C_{min} (ng/mL)	21.74 ± 8.99	17.01 ± 6.24	14.4 ± 5.88	11.41 ± 4.04	9.92 ± 4.05	8.14 ± 2.88	7.32 ± 2.99	6.2 ± 2.2	5.71 ± 2.31	4.98 ± 1.78	4.68 ± 1.86	4.18 ± 1.51	3.99 ± 1.57	3.7 ± 1.37	3.46 ± 1.2	3.41 ± 1.26
	$AUC_{0\text{-}24ss}$ (ng/mL.h)	639.06 ± 234.63	516.44 ± 170.6	422.5 ± 153.11	343.7 ± 110.07	290.11 ± 105.41	243.79 ± 77.63	213.42 ± 77.74	185.06 ± 59.37	166.27 ± 60.09	148.15 ± 48.07	136.22 ± 48.48	124.19 ± 41.24	116.05 ± 40.94	109.81 ± 37.13	101.19 ± 31.32	101.03 ± 34.21
	% C_{max} > 67 ng/mL	0	0	0	0	0	0	0	0	0	0	0	0	0	0	0	0
	1 ng/mL < % C_{max} < 57.2 ng/mL	100	100	100	100	100	100	100	100	100	100	100	100	100	100	100	100
5.00 mg OD	C_{max} (ng/mL)	42.66 ± 13.93	35.48 ± 11.18	27.91 ± 8.99	23.29 ± 7.07	18.99 ± 6.16	16.38 ± 4.88	13.9 ± 4.52	12.37 ± 3.72	10.79 ± 3.48	9.87 ± 3	8.82 ± 2.8	8.25 ± 2.6	7.51 ± 2.36	7.29 ± 2.34	6.57 ± 1.84	6.69 ± 2.17
	C_{min} (ng/mL)	29.83 ± 12.25	23.31 ± 8.6	19.6 ± 7.98	15.51 ± 5.52	13.43 ± 5.47	11 ± 3.91	9.87 ± 4.02	8.36 ± 2.97	7.68 ± 3.1	6.7 ± 2.39	6.29 ± 2.5	5.62 ± 2.03	5.35 ± 2.11	4.97 ± 1.85	4.64 ± 1.61	4.58 ± 1.69
	$AUC_{0\text{-}24ss}$ (ng/mL.h)	873.77 ± 320	704.83 ± 234.49	573.83 ± 207.59	465.96 ± 150.18	392.04 ± 142.34	329.09 ± 105.29	287.49 ± 104.69	249.14 ± 80.22	223.52 ± 80.77	199.09 ± 64.8	182.86 ± 65.08	166.68 ± 55.49	155.65 ± 54.93	147.25 ± 49.86	135.62 ± 41.98	135.42 ± 45.92
	% C_{max} > 67 ng/mL	6.25	0	0	0	0	0	0	0	0	0	0	0	0	0	0	0
	1 ng/mL < % C_{max} < 57.2 ng/mL	83.33	98.08	97.87	100	100	100	100	100	100	100	100	100	100	100	100	100
6.25 mg OD	C_{max} (ng/mL)	54.39 ± 17.75	45.17 ± 14.3	35.42 ± 11.41	29.51 ± 9.01	24.01 ± 7.79	20.68 ± 6.19	17.52 ± 5.7	15.59 ± 4.7	13.58 ± 4.38	12.41 ± 3.79	11.09 ± 3.52	10.37 ± 3.28	9.43 ± 2.97	9.16 ± 2.95	8.25 ± 2.32	8.4 ± 2.73
	C_{min} (ng/mL)	38.28 ± 15.62	29.9 ± 11.09	25 ± 10.12	19.75 ± 7.07	17.03 ± 6.92	13.95 ± 4.97	12.47 ± 5.08	10.56 ± 3.77	9.69 ± 3.91	8.45 ± 3.03	7.92 ± 3.14	7.07 ± 2.57	6.74 ± 2.65	6.25 ± 2.32	5.83 ± 2.02	5.76 ± 2.13
	$AUC_{0\text{-}24ss}$ (ng/mL.h)	1118 ± 407.84	900.7 ± 301.5	730.04 ± 263.51	591.9 ± 191.89	496.48 ± 180.08	416.35 ± 133.81	362.98 ± 132.12	314.38 ± 101.59	281.64 ± 101.76	250.79 ± 81.88	230.12 ± 81.9	209.73 ± 69.99	195.7 ± 69.07	185.09 ± 62.77	170.4 ± 52.76	170.16 ± 57.78
	% C_{max} > 67 ng/mL	16.67	5.77	2.13	0	0	0	0	0	0	0	0	0	0	0	0	0
	1 ng/mL < % C_{max} < 57.2 ng/mL	68.75	76.92	95.74	100	100	100	100	100	100	100	100	100	100	100	100	100
7.50 mg OD	C_{max} (ng/mL)	66.48 ± 21.65	55.15 ± 17.54	43.11 ± 13.87	35.87 ± 11	29.12 ± 9.45	25.07 ± 7.53	21.2 ± 6.9	18.85 ± 5.7	16.41 ± 5.29	14.99 ± 4.59	13.39 ± 4.25	12.52 ± 3.96	11.37 ± 3.58	11.04 ± 3.56	9.94 ± 2.8	10.12 ± 3.29
	C_{min} (ng/mL)	47.08 ± 19.05	36.76 ± 13.68	30.57 ± 12.32	24.12 ± 8.67	20.73 ± 8.4	16.96 ± 6.07	15.13 ± 6.15	12.81 ± 4.58	11.73 ± 4.73	10.22 ± 3.67	9.57 ± 3.8	8.55 ± 3.11	8.13 ± 3.2	7.54 ± 2.81	7.04 ± 2.44	6.95 ± 2.57
	$AUC_{0\text{-}24ss}$ (ng/mL.h)	1370.97 ± 497.56	1103.59 ± 371.32	890.9 ± 320.68	721.4 ± 235.13	603.34 ± 218.55	505.54 ± 163.17	439.85 ± 160.01	380.78 ± 123.46	340.65 ± 123.05	303.26 ± 99.29	277.98 ± 98.93	253.31 ± 84.73	236.2 ± 83.37	223.35 ± 75.85	205.52 ± 63.65	205.25 ± 69.78
	% C_{max} > 67 ng/mL	35.42	30.77	6.38	0	0	0	0	0	0	0	0	0	0	0	0	0
	1 ng/mL < % C_{max} < 57.2 ng/mL	31.25	48.08	87.23	98.11	97.87	100	100	100	100	100	100	100	100	100	100	100
8.75 mg OD	C_{max} (ng/mL)	78.91 ± 25.61	65.4 ± 20.88	35.87 ± 11	42.38 ± 13.06	34.33 ± 11.13	29.53 ± 8.91	24.94 ± 8.11	22.16 ± 6.72	19.27 ± 6.22	17.6 ± 5.4	15.7 ± 4.99	14.68 ± 4.66	13.33 ± 4.2	12.94 ± 4.18	11.65 ± 3.28	11.86 ± 3.86
	C_{min} (ng/mL)	56.2 ± 22.54	43.87 ± 16.38	24.12 ± 8.67	28.63 ± 10.33	24.52 ± 9.9	20.05 ± 7.19	17.84 ± 7.24	15.1 ± 5.41	13.8 ± 5.56	12.03 ± 4.33	11.25 ± 4.46	10.05 ± 3.66	9.55 ± 3.75	8.85 ± 3.3	8.26 ± 2.86	8.15 ± 3.02
	$AUC_{0\text{-}24ss}$ (ng/mL.h)	1631.94 ± 588.67	1313.04 ± 443.61	721.4 ± 235.13	854.31 ± 279.81	712.54 ± 257.7	596.61 ± 193.34	518.08 ± 188.33	448.31 ± 145.84	400.51 ± 144.62	356.48 ± 117.04	326.44 ± 116.15	297.44 ± 99.71	277.14 ± 97.83	262 ± 89.1	240.98 ± 74.64	240.68 ± 81.93
	% C_{max} > 67 ng/mL	70.83	51.92	14.77	1.89	2.13	0	0	0	0	0	0	0	0	0	0	0
	1 ng/mL < % C_{max} < 57.2 ng/mL	22.92	38.46	72.34	84.91	95.75	100	100	100	100	100	100	100	100	100	100	100
10.00 mg OD	C_{max} (ng/mL)	91.64 ± 29.62	75.91 ± 24.3	59.04 ± 18.94	49.02 ± 15.16	39.64 ± 12.85	34.06 ± 10.31	28.73 ± 9.35	25.52 ± 7.77	22.17 ± 7.16	20.24 ± 6.23	18.05 ± 5.73	16.87 ± 5.36	15.31 ± 4.82	14.85 ± 4.8	13.37 ± 3.76	13.62 ± 4.44
	C_{min} (ng/mL)	65.6 ± 26.06	51.23 ± 19.16	42.22 ± 16.83	33.27 ± 12.05	28.39 ± 11.43	23.21 ± 8.35	20.6 ± 8.34	17.43 ± 6.26	15.91 ± 6.39	13.86 ± 5	12.95 ± 5.13	11.56 ± 4.22	10.98 ± 4.31	10.18 ± 3.79	9.49 ± 3.28	9.37 ± 3.47
	$AUC_{0\text{-}24ss}$ (ng/mL.h)	1900.24 ± 680.76	1528.62 ± 518.06	1225.57 ± 438.09	990.5 ± 325.84	824.02 ± 297.45	689.51 ± 224.3	597.64 ± 217.05	516.97 ± 168.7	461.22 ± 166.46	410.44 ± 135.12	375.48 ± 133.57	342.09 ± 114.93	318.52 ± 112.43	301.06 ± 102.52	276.79 ± 85.74	276.47 ± 94.22
	% C_{max} > 67 ng/mL	77.08	61.54	25.53	13.21	4.26	0	0	0	0	0	0	0	0	0	0	0
	1 ng/mL < % C_{max} < 57.2 ng/mL	10.42	32.69	53.19	67.93	93.62	100	97.87	100	100	100	100	100	100	100	100	100

2 3 4 5 6 7 8 9 10 11 12 13 14 15 16 17 18
Age

Figure S15. Summary of pharmacokinetic parameters at steady-state in paediatric obesity from 2 to 18 years old administered with fixed dose. C_{max}, maximum concentration; C_{min}, minimum concentration; $AUC_{0\text{-}24ss}$, area-under-the-curve at steady-state; 67 ng/mL, toxic level; 1 ng/mL to 57.2 ng/mL, therapeutic concentration.

References

ABITBOL, C. L., CHANDAR, J., RODRIGUEZ, M. M., BERHO, M., SEEHERUNVONG, W., FREUNDLICH, M. & ZILLERUELO, G. 2009. Obesity and preterm birth: additive risks in the progression of kidney disease in children. *Pediatr Nephrol,* 24**,** 1363-70.

BELO, L., NASCIMENTO, H., KOHLOVA, M., BRONZE-DA-ROCHA, E., FERNANDES, J., COSTA, E., CATARINO, C., AIRES, L., MANSILHA, H. F., ROCHA-PEREIRA, P., QUINTANILHA, A., REGO, C. & SANTOS-SILVA, A. 2014. Body fat percentage is a major determinant of total bilirubin independently of UGT1A1*28 polymorphism in young obese. *PLoS One,* 9**,** e98467.

CACCIARI, E., BALSAMO, A., PALARETI, G., CASSIO, A., ARGENTO, R., POGGI, M., TASSONI, P., CICOGNANI, A., TACCONI, M., PASCUCCI, M. G. & ET AL. 1988. Haemorheologic and fibrinolytic evaluation in obese children and adolescents. *Eur J Pediatr,* 147**,** 381-4.

CENTERS FOR DISEASE CONTROL AND PREVENTION (CDC). 2017. *Clinical Growth Charts* [Online]. Available: https://www.cdc.gov/growthcharts/clinical_charts.htm [Accessed 18 January 2023].

CORREIA-COSTA, L., SCHAEFER, F., AFONSO, A. C., BUSTORFF, M., GUIMARAES, J. T., GUERRA, A., BARROS, H. & AZEVEDO, A. 2016. Normalization of glomerular filtration rate in obese children. *Pediatr Nephrol,* 31**,** 1321-8.

DUZOVA, A., YALCINKAYA, F., BASKIN, E., BAKKALOGLU, A. & SOYLEMEZOGLU, O. 2013. Prevalence of hypertension and decreased glomerular filtration rate in obese children: results of a population-based field study. *Nephrol Dial Transplant,* 28 Suppl 4**,** iv166-71.

ELHAG, W., EL ANSARI, W., ABDULRAZZAQ, S., ABDULLAH, A., ELSHERIF, M. & ELGENAIED, I. 2018. Evolution of 29 Anthropometric, Nutritional, and Cardiometabolic Parameters Among Morbidly Obese Adolescents 2 Years Post Sleeve Gastrectomy. *Obes Surg,* 28**,** 474-482.

FERRARI, M., CUENCA-GARCIA, M., VALTUENA, J., MORENO, L. A., CENSI, L., GONZALEZ-GROSS, M., ANDROUTSOS, O., GILBERT, C. C., HUYBRECHTS, I., DALLONGEVILLE, J., SJOSTROM, M., MOLNAR, D., DE HENAUW, S., GOMEZ-MARTINEZ, S., DE MORAES, A. C., KAFATOS, A., WIDHALM, K., LECLERCQ, C. & GROUP, H. S. 2015. Inflammation profile in overweight/obese adolescents in Europe: an analysis in relation to iron status. *Eur J Clin Nutr,* 69**,** 247-55.

GERHART, J. G., CARRENO, F. O., EDGINTON, A. N., SINHA, J., PERRIN, E. M., KUMAR, K. R., RIKHI, A., HORNIK, C. P., HARRIS, V., GANGULY, S., COHEN-WOLKOWIEZ, M., GONZALEZ, D. & BEST PHARMACEUTICALS FOR CHILDREN ACT-PEDIATRIC TRIALS NETWORK STEERING, C. 2022. Development and Evaluation of a Virtual Population of Children with Obesity for Physiologically Based Pharmacokinetic Modeling. *Clin Pharmacokinet,* 61**,** 307-320.

GIBSON, R. S., BAILEY, K. B., WILLIAMS, S., HOUGHTON, L., COSTA-RIBEIRO, H. C., MATTOS, A. P., BARRETO, D. L. & LANDER, R. L. 2014. Tissue iron deficiency and adiposity-related inflammation in disadvantaged preschoolers from NE Brazil. *Eur J Clin Nutr,* 68**,** 887-91.

GOKNAR, N., OKTEM, F., OZGEN, I. T., TORUN, E., KUCUKKOC, M., DEMIR, A. D. & CESUR, Y. 2015. Determination of early urinary renal injury markers in obese children. *Pediatr Nephrol,* 30**,** 139-44.

JEONG, H. R., SHIM, Y. S., LEE, H. S. & HWANG, J. S. 2021. Hemoglobin and hematocrit levels are positively associated with blood pressure in children and adolescents 10 to 18 years old. *Sci Rep,* 11**,** 19052.

KILIC, E., OZER, O. F., EREK TOPRAK, A., ERMAN, H., TORUN, E., KESGIN AYHAN, S., CAGLAR, H. G., SELEK, S. & KOCYIGIT, A. 2016. Oxidative Stress Status in Childhood Obesity: A Potential Risk Predictor. *Med Sci Monit,* 22**,** 3673-3679.

MARGINEAN, C. O., CLAUDIA, B., CARMEN, D., MARIA, P. A., SEPTIMIU, V. & CLAUDIU, M. 2014. The role of IL-6 572 C/G, 190 C/T, and 174 G/C gene polymorphisms in children's obesity. *Eur J Pediatr,* 173**,** 1285-96.

MARGINEAN, C. O., MARGINEAN, C., VOIDAZAN, S., MELIT, L., CRAUCIUC, A., DUICU, C. & BANESCU, C. 2016. Correlations Between Leptin Gene Polymorphisms 223 A/G, 1019 G/A, 492 G/C, 976 C/A, and Anthropometrical and Biochemical Parameters in Children With Obesity: A Prospective Case-Control Study in a Romanian Population-The Nutrichild Study. *Medicine (Baltimore),* 95**,** e3115.

MARGINEAN, C. O., MELIT, L. E., GHIGA, D. V. & MARGINEAN, M. O. 2019. Early Inflammatory Status Related to Pediatric Obesity. *Front Pediatr,* 7**,** 241.

MARGINEAN, C. O., MELIT, L. E., HUTANU, A., GHIGA, D. V. & SASARAN, M. O. 2020. The adipokines and inflammatory status in the era of pediatric obesity. *Cytokine,* 126**,** 154925.

ONI, O., OREKOYA, O. & BAMJI, M. 2021. Prevalence of Disease Conditions and Laboratory Findings in Obese Children: A Decade Analysis of National Health and Nutrition Examination Survey 2005-2014. *Pediatrics,* 147.

PANICHSILLAPHAKIT, E., SUTEEROJNTRAKOOL, O., PANCHAROEN, C., NUCHPRAYOON, I. & CHOMTHO, S. 2021. The Association between Hepcidin and Iron Status in Children and Adolescents with Obesity. *Journal of Nutrition and Metabolism,* 2021.

SOBIESKA, M., GAJEWSKA, E., KALMUS, G. & SAMBORSKI, W. 2013. Obesity, physical fitness, and inflammatory markers in Polish children. *Med Sci Monit,* 19**,** 493-500.

WORLD HEALTH ORGANISATION (WHO). 2021. *Obesity and overweight* [Online]. Available: https://www.who.int/news-room/fact-sheets/detail/obesity-and-overweight [Accessed 18 January 2023].

YU, L., LI, Y., ZHANG, Q., ZHU, L., DING, N., ZHANG, B., ZHANG, J., LIU, W., LI, S. & ZHANG, J. 2021. Association between dietary essential amino acids intake and metabolic biomarkers: influence of obesity among Chinese children and adolescents. *Amino Acids,* 53**,** 635-644.
